# A simple routine for quantitative analysis of light and dark kinetics of photochemical and non-photochemical quenching of chlorophyll fluorescence in intact leaves

**DOI:** 10.1007/s11120-015-0097-x

**Published:** 2015-03-05

**Authors:** Wim Vredenberg

**Affiliations:** 1 Department of Plant Physiology, Wageningen University and Research, Wageningen, The Netherlands; 2 Department of Plant Physiology, Wageningen University and Research, Droevendaalsesteeg 1, 6708 PB Wageningen, The Netherlands

**Keywords:** Chlorophyll fluorescence kinetics, Quenching mechanisms, System analysis, TSTM, OJIP, Views

## Abstract

Paper describes principles and application of a novel routine that enables the quantitative analysis of the photochemical O–J phase of the variable fluorescence *F*_v_ associated with the reversible photo-reduction of the secondary electron acceptor Q_A_ of photosystem II (PSII) in algae and intact leaves. The kinetic parameters that determine the variable fluorescence *F* ^PP^(*t*) associated with the release of photochemical quenching are estimated from 10 µs time-resolved light-on and light-off responses of *F*_v_ induced by two subsequent light pulses of 0.25 (default) and 1000 ms duration, respectively. Application of these pulses allows estimations of (i) the actual value of the rate constants *k*_L_ and *k*_AB_ of the light excitation (photoreduction of Q_A_) and of the dark re-oxidation of photoreduced Q_A_ (${\text{Q}}_{\text{A}}^{ - }$), respectively, (ii) the actual maximal normalized variable fluorescence [*nF*_v_] associated with 100 % photoreduction of Q_A_ of open RCs, and (iii) the actual size *β* of RCs in which the re-oxidation of ${\text{Q}}_{\text{A}}^{ - }$ is largely suppressed (Q_B_-nonreducing RC with *k*_AB_ ~ 0). The rate constants of the dark reversion of *F*v associated with the release of photo-electrochemical quenching *F* ^PE^ and photo-electric stimulation *F* ^CET^ in the successive J–I and I–P parts of the thermal phase are in the range of (100 ms)^−1^ and (1 s)^−1^, respectively. The kinetics of fluorescence changes during and after the I–P phase are given special attention in relation to the hypothesis on the involvement of a Δ*µ*_H+_-dependent effect during this phase and thereafter. Paper closes with author’s personal view on the demands that should be fulfilled for chlorophyll fluorescence methods being a correct and unchallenged signature of photosynthesis in algae and plants.

## Introduction

The time pattern of variable chlorophyll a (chla) fluorescence of alga and plant leaves (chla fluorescence induction) in an actinic light pulse provides valuable information on properties and characteristics of the photosynthetic processes that are initiated by the light. Amongst those are (i) generation and decay of trans- and inner membrane electric fields associated with primary charge separation in the photochemical systems PSI and PSII, (ii) photochemical reduction of the primary electron acceptor pair [PheQ_A_] with pheophytin (Phe) and Q_A_ acting as primary and secondary electron acceptors and fluorescence quenchers, respectively, (iii) secondary processes that are coupled to electron transport in the photosynthetic transport chains, that among others lead to generation and dissipation of a trans-thylakoid electrochemical proton gradient (Δ*µ*_H_) which powers ATP synthesis and transmembrane ion fluxes.

The fluorescence induction pattern *F*(*t*) in a dark-adapted leaf or algal suspension shows at high actinic intensities a poly-phasic so-called OJIP increase in variable fluorescence *F*_v_(*t*) from an initial *F*_o_ level at O toward a maximal *F*_m_ level at *P*. The *F*_o_ to *F*_m_ rise in the light usually covers a time span of five decades from 10 μs to 1 s and is followed in prolonged illumination by a so-called PSMT decay in a time range extending to several minutes (Govindjee 2004; Papageorgiou et al. 2007). The labels in the OJIPSMT (or Kautsky) fluorescence induction curve mark the intercept of subsequent response phases in which the apparent rate of fluorescence increase or decrease is different.

The OJIP part of the Kautsky fluorescence induction curve has received ample attention from distinctly different viewpoints. The first group is primarily focussed upon a mathematical analysis and presentation of the characteristic shape of the constituting O–J, J–I, and I–P components (Pospìsil and Dau 2000; Boisvert et al. 2006; Antal and Rubin 2008; Joly and Carpentier 2009). I will denote it here with the math-fit-test (MFT). MFT leads to the fitting of an OJIP curve with the sum of three exponential functions, including those with a coefficient accommodating the sigmoidal character of the distinguishable phases (Joly and Carpentier 2009). MFT is hampered by the fact that none of its parameters bears a simple relation to those of the photochemical and non-photochemical reactions that are at the basis of and responsible for the bioenergetic performance of the photosynthetic system under study. The second class uses the so called JIP test introduced by Reto Strasser and his coworkers (Strasser et al. 1995, 2004; Stirbet and Govindjee 2012). The JIP test is a systematic method and practical tool to obtain quick information, particularly on PSII, from the OJIP induction curve on various (possibilities of) effects on photosynthesis. The information is gathered and estimated in this test from the fluorescence emission data at a limited number, usually 6, of (time) locations within the monitoring period of the fluorescence emission induced by a fixed actinic light intensity, usually ~3000 µmol photons.m^−2^ s^−1^. The JIP test is, among others, based on the assumption that (i) the maximum fluorescence *F*_m_ is exclusively associated with 100 % reduction of the primary quinone acceptor Q_A_ and (ii) *F*_m_ can be reached, for instance in the presence of a herbicide like DCMU, in one single-saturating light flash (STF). The JIP test has found many applications in eco-physiological research dealing with the effect of several environmental stress forms on plant performance (see for a recent review Guo and Tan 2015). The third category uses system approaches in which the variable fluorescence is analyzed in terms of kinetic parameters of primary and associated photosynthetic reactions linked to *F*_v_. One of these is aimed at availability and use of a fluorescence induction algorithm (FIA) with manageable expressions for the photochemical and non photochemical (thermal) components of the variable fluorescence during the OJIP traject. Its application is called FIA methodology. The constituting mathematical expressions are based on analysis and solutions of the kinetic equations of the underlying reactions in terms of identifiable reaction parameters, amongst which the actinic intensity in the range between 30 and 3000 µmol photons.m^−2^ s^−1^. The FIA methodology is conceptually different from the alternate approaches in a sense that it is based on the concept (Vredenberg 2000) of the three-state trapping model (TSTM) and as such not limited by the disputable constraint (Stirbet and Govindjee 2012) that 100 % reduction of the primary quinone acceptor Q_A_ is required and sufficient for reaching the maximum fluorescence *F*_m_.

The time scale patterns of the OJIP rise in algae and leaves at light intensities of about 1000 μmol quanta m^−2^ s^−1^ commonly shows an initial exponential O–J increase toward a quasi-stationary level J within a few ms and followed by two sequential S-shaped J–I and I–P rises that are completed within 30 and 500 ms, respectively. The patterns show intercept levels at J, I, and P with 2.5 < *F*_J_/*F*_o_ < 3.5, 4.5 < *F*_P_/*F*_o_) < 5.5, and *F*_I_ ~ 10 % below *F*_P_. The different sensitivities of the OJ and JIP responses to alterations in among others light intensity, temperature or PSII-inhibiting herbicides has led to their distinction as the photochemical (OJ) and a non-photochemical thermal phase (JIP) (for literature survey and reviews see Samson et al. 1999; Stirbet and Govindjee 2012; Schansker et al. 2011).

The interpretation of the OJIP induction profile in terms of reactions and processes that are involved, is under continuing debate (Stirbet and Govindjee 2012; Vredenberg et al. 2012; Schansker et al. 2013). A large variety of simulation models for the OJIP induction curve has been presented that describe the variable fluorescence at a given light intensity (excitation rate) in relation to reaction center closure (Stirbet et al. 1998; Vredenberg 2000; Strasser et al. 2004; Kramer et al. 2004; Zhu et al. 2005; Lazár and Schansker 2009; Belyaeva et al. 2008). RC closure in most of these concepts is assumed to be exclusively due to single-photon trapping in the RC of PSII and the stabilization of an electron at its acceptor side as reflected by the light-driven reduction of the Q_A_. Photoreduction of Q_A_ is thought, following the interpretation of Duysens and Sweers (1963), to release the quenching properties of the oxidized form of Q_A_. Fluorescence changes elicited with (sub-)ns excitations have indicated that the oxidized primary donor of PSII (P680^+^) quenches the fluorescence as well (Butler 1972; Mauzerall 1972). A conceptually different so called double-hit three state trapping model (TSTM) has been proposed (Vredenberg 2000, 2004; Vredenberg and Prasil 2009). This takes into account, as outlined in detail in the literature (Vredenberg et al. 2009; Vredenberg et al. 2012) that RC-closure, i.e., the increase in variable fluorescence, is not exclusively and necessarily caused by the photochemical reduction of Q_A_, but is also promoted by photo-electrochemical and electrical events in the vicinity of the membrane bound RC. The following characteristic differences between the concept of the ‘classic’ single-hit trapping models and that of TSTM are (i) operation of a double-hit trapping mechanism in TSTM in which the primary PSII electron acceptor pair [PheQ_A_] of open RCs acts as a competent two electron trap, (ii) two successive single-turnover excitations are required for semi-closure [PheQ_A_]^−1^ and subsequent closure [PheQ_A_]^2−^ of the RC (Vredenberg 2000, 2011), and (iii) semi-closure of all open RCs [inducible in chloroplasts by a saturating single-turnover flash (STF)] is accompanied by a normalized variable fluorescence $nF_{\text{v}}^{\text{STF}} = (F_{\text{m}}^{\text{S}} {\text{TF}} - F_{\text{o}} )/(F_{\text{o}} ) \sim 2;$ full closure [inducible by repetitive STFs or by 250 ms fluorescence saturating pulses (SP)] results in an approximate doubling of the normalized variable fluorescence $nF_{\text{v}}^{\text{SP}} = {{F_{\text{m}}^{\text{SP}} - F_{\text{o}} }}/{{F_{\text{o}} }} \sim 4$, which suggests a normalized variable fluorescence per trapped electron *nF*_v_ ~ 2 (Vredenberg et al. 2012), (iv) chlorophyll fluorescence yield is sensitive to electrochemical changes, in particular to that of the transmembrane electrochemical gradient of protons (Δ*µ*_H+_) which are (is) coupled to linear and cyclic electron transport between the photosystems and around PSI, respectively, and (v) the concept of so called ‘inactive’ RCs is in TSTM substituted by a dynamic heterogeneity of Q_B_-reducing and Q_B_-nonreducing RCs (Chylla and Whitmarsh 1989; Lavergne and Leci 1993; Tomek et al. 2003; Vredenberg et al. 2006). The conclusion that the STF-induced saturation of photochemical quenching is associated with approx. 50 % of the SP-induced maximal variable fluorescence *F*_m_ has been confirmed in a recent study with the alga Chlorella (Klughammer and Schreiber 2015). An alternate interpretation of the non photochemical thermal JIP phase has been proposed in which the fluorescence de-quenching reaction is presumed to be due to a light-driven conformational change in PSII (Schansker et al. 2011).

This paper gives illustrations and kinetic analyses of the light-on and light-off responses of the variable fluorescence $F_{\text{v}} \left( t \right)/F_{\text{o}} \left[ { = \, F\left( t \right)/F_{\text{o}} {-}{ 1}} \right]$ in intact leaves and algae upon light pulses variable in duration and intensity. Analysis of the time responses is based on the solution of the equation for a light–dark reversible transfer of an RC with Q_A_ toward one with ${\text{Q}}_{\text{A}}^{ - }$. Application of pulses in the time range between 0.25 and 1 ms enables estimations of (i) the actual value of the rate constants *k*_L_ and *k*_AB_ of the light excitation (photoreduction of Q_A_) and of the dark re-oxidation of photoreduced Q_A_ (${\text{Q}}_{\text{A}}^{ - }$), respectively, (ii) the actual maximal normalized variable fluorescence *nF*_v_ associated with 100 % photoreduction of Q_A_ of open RCs, (iii) the actual size *β* of RCs in which the re-oxidation of ${\text{Q}}_{\text{A}}^{ - }$ is largely suppressed (Q_B_-nonreducing RC with *k*_AB_ ~ 0), and (iv) a distinct decrease with pulse duration of the initial rate of the fluorescence recovery (re-quenching) at light off, presumably indicating the pH dependence of *k*_AB_. The results give strong support for the hypothesis that the photochemical O–J phase in the 0.01–2 ms time range of the OJIP induction curve is, for the major part, caused by the variable fluorescence *F* ^PP^(*t*) associated exclusively with the primary photoreduction of Q_A_. A simple routine program for estimating the actual kinetic parameters of the photochemical fluorescence induction phase in intact leaves and algae is outlined and is available upon request. Pulses in the time range covering the J–I phase show responses with, for the major components, rate constants in the range of (10 ms)^−1^ and (100 ms)^−1^ in the light and dark, respectively. The characteristics of those in the range of the I–P phase add to the evidence that the variable fluorescence *F* ^CET^(*t*) in this phase originates from the build-up of the proton motive force by the light-driven proton pump coupled to cyclic electron transport around PSI.

The paper is concluded with a personal view on the present status of chlorophyll fluorescence in relation to its potency for being ‘a signature of photosynthesis’. It expresses my feeling on the urgent need for coming to an agreement on the controversial views on the as yet unsolved problem whether or not the closure of the photosynthetic reaction center and its associated maximal increase in variable fluorescence toward *F*_m_ can be accomplished only by the photochemical conversion of Q_A_. The answer to this question has far reaching consequences for the validity of some of the conclusions on the photosynthetic performance and characteristics of intact leaves and algae obtained with current fluorescence techniques.

## Materials and methods

*Nannochloropsis* sp. (CCAP 211/78) cells were grown in June in an outdoor tubular fence-type photo bioreactor at a dedicated facility of the Wageningen University, (<http://www.algaeparc.com>). Tube diameter was 4.6 cm and the cells were grown in seawater enriched with nutrients at pH 7.5 and at a temperature between 25 and 30 °C. Cell concentration in the photobioreactor was maintained constant at 1.5 g dry weight per liter by continuous reactor dilution. This concentration corresponds to approximately 10 µg chl ml^−1^; 4 ml samples were directly transferred to 1 × 1 cm cuvettes in the sample holder of the measuring device. Young leaves of *Arum italiensis, Rosea gislaine*, and *Kalanchoë* were collected from plants in the home garden; spinach leaves from a fresh batch were from a local supermarket. Leaves were positioned in the leaf holder of the measuring device.

Fluorescence experiments were done using the modulated chlorophyll fluorometer OS1p (Opti-Science Ltd, Hudson, USA) in its so-called FIA-OJIP routine (Vredenberg et al. 2013). Light-on and light-off kinetics of the variable fluorescence in light pulses variable in length from 0.25 ms to tens of seconds, and of intensity in the range between 50 and 5000 µmol photons.m^−2^ s^−1^ can be monitored. The time resolution during a light and dark period is variably programmable at values from 10 µs to 1 s. Special attention is given to application of short saturating pulses (sSPs) in the time range between 250 and 1000 µs. The experimental traces in general represent the averages of five samples. Curve fitting of the experimental quenching responses was done with application of proper routines provided by Excel software.

## Theoretical aspects

### Light-on and light-off kinetics of variable fluorescence in cells and leaves; photochemical phase

The photochemical-driven dark-reversible change in the fluorescence yield of the PSII antenna’s is variable between *F*_o_ and *F*_m_ for centers in which the PSII electron acceptor side denoted with [PheQ_A_] is oxidized (open centers) and single reduced ([PheQ_A_]^1−^, semi-closed), respectively. The monitoring of light-on and light-off kinetics has been shown to enable a quantitative analysis of alterations in photochemical quenching of PSII fluorescence under variable conditions (Vredenberg and Prasil 2013). The increase in the variable chlorophyll fluorescence $F_{\text{v}} \left( { = \frac{{F\left( t \right) - F_{\text{o}} }}{{F_{\text{o}} }}} \right)$ at the onset of light is attributed, as first demonstrated by Duysens and Sweers (1963), to the de-quenching of PS II antenna fluorescence associated with energy trapping and stabilization in a PSII reaction center leading at the acceptor side to photoreduction of Q_A_.

Under conditions at which effects of intersystem energy transfer (connectivity) between photosynthetic units [see for a review (Stirbet 2013)] and of donor side quenching in PS II by the secondary electron donor of PS II ($Y_{\text{z}}^{ + }$) are negligible, the photochemical-driven initial *F*_v_ increase is theoretically predicted to be exponential (Vredenberg 2008a). The reaction rate at the onset of illumination is determined by the light excitation rate *k*_L_, and has been shown to vary linearly with light intensity. For a great variety of plant species incident PAR intensity at the leaf surface of ~2000 µmol quanta.m^−2^ s^−1^ is found to correspond globally with a value *k*_L_ ~ 1 ms^−1^.

The reversal of the photochemically generated signal at light off (dark decay of variable fluorescence) is the consequence of (re-) quenching associated with re-oxidation of the reduced electron acceptor ${\text{Q}}_{\text{A}}^{ - }$ by secondary electron acceptors. This light-independent oxidation proceeds in dark-adapted samples with a rate constant *k*_AB_ that has been reported to be in the range between 2 and 5 ms^−1^ (Robinson and Crofts 1983).

The time pattern of the photochemical de- and re-quenching of the fluorescence by Q_A_ during and after actinic illumination is predicted by the analytical solution of the ordinary differential equations (ODE’s) for the reversible photoreduction of Q_A_ (Vredenberg 2011). Briefly, the kinetic analysis of the reaction that describes the photochemical reduction of Q_A_ gives a quantitative expression for the fraction *q* ^dsq^ of centers that has become photochemically closed in the light at time *t*, with

1 $$q_{0}^{\text{dsq}} \left( t \right) = \frac{{k_{\text{L}} }}{{k_{\text{L}} + k_{\text{AB}} }}\; \times \;[1 - e^{{ - \left( {k_{\text{L}} + k_{\text{AB}} } \right)t}} ],$$

where the superscript ^dsq^ refers to the condition that donor-side quenching (by $Y_{\text{z}}^{ + }$) is considered to be negligible (Vredenberg 2011) and the subscript _0_ to that of a homogeneous system in which the fraction of the so-called Q_B_-nonreducing RCs, in which *k*_AB_ ~ 0, is zero. Accordingly,

1a $$q_{\beta }^{\text{dsq}} \left( t \right) = [1 - e^{{ - k_{\text{L}} \cdot t}} ]$$

For a heterogeneous system with a *β*-fraction of Q_B_-nonreducing RCs

2 $$q^{\text{dsq}} \left( t \right) = \left( {1 - \beta } \right) \cdot q_{0}^{\text{dsq}} \left( t \right) + \beta \,\times\, q_{\beta }^{\text{dsq}} \left( t \right)$$

The ‘re-opening’ recovery of the fractions in the dark, after light off at *t* = *t*_0_, follows the exponential function

3 $$q_{\text{d}}^{\text{dsq}} \left( t \right) = q^{\text{dsq}} (t_{0} )\; \times \;e^{{ - \left( {k_{\text{AB}} } \right)t}} ,$$

where the subscript _d_ refers to darkness. The variable fluorescence *F* ^PP1^(*t*) associated with the photochemical-dependent de- and re-quenching during and after illumination is given by

4 $$F^{\text{PP1}} \left( t \right) = nF_{\text{v}} \,\times\, q^{\text{dsq}} (t),$$

where *nF*_v_ is the maximal fluorescence $F_{\text{m}}^{\text{PP}}$ when Q_A_ is 100 % (photo-) reduced, i.e., when all RCs are photochemically closed and *q* ^dsq^ = 1.

Equations 1–4 demonstrate the inadequacy of the earlier mentioned MTF to fit the O–J phase of the OJIP curve with a single exponential of the form $F = F_{\text{o}} + \, A_{\text{O - J}} \left[ { 1- { \exp }\left( { - k_{\text{O - J}} \cdot t} \right)} \right]$ (Joly and Carpentier 2009). MFT will not provide handsome information on the processes that are responsible for the O–J rise during fluorescence induction.

It has been shown (Vredenberg et al. 2006) that the *β*-fraction of Q_B_-nonreducing RCs, has a non-zero efficiency *Φ* for transiently trapping a second electron causing the transient double reduction of the PSII acceptor side in these RCs. This is associated with a variable fluorescence

5 $$F^{\text{PP2}} \left( t \right) = \beta \cdot nF_{\text{v}} \cdot q_{\beta }^{\text{dsq}} \left( t \right) \cdot (1 - e^{{ - \emptyset \cdot k_{\text{L}} \cdot t}} ) \cdot e^{{ - k_{{2{\text{AB}}}} \cdot t}}.$$

In which *Φ* is the electron trapping efficiency in the fraction with reduced Q_B_-nonreducing RCs and *k*_2AB_ the re-oxidation rate constant of the double-reduced acceptor pair (Vredenberg and Prasil 2009; Vredenberg 2011). It is noteworthy that, according to Eq. 5, d*F* ^PP2^(*t*)/d*t* = 0 at *t* = 0, which causes an S-shaped *F* ^PP2^(*t*)-response. The analytical solution for the variables fluorescence associated with the primary photochemical events is obtained after summation of Eqs. 4 and 5

6 $$F^{\text{PP}} \left( t \right) = F^{\text{PP1}} \left( t \right) + F^{\text{PP2}} \left( t \right).$$

It easily follows from Eqs. 1–4, that *F* ^PP1^(*t*) reaches for *t* ≫ (*k*_L_ + *k*_AB_)^−1^ a time-independent equilibrium steady state $F_{\text{ss}}^{\text{PP1}}$ in the light equal to

6(a $$F_{\text{ss}}^{\text{PP1}} = nF_{\text{v}} \cdot \frac{{k_{\text{L}} }}{{k_{\text{L}} + k_{\text{AB}} }}.$$

Similarly one obtains, for *t* ≫ (*k*_2AB_)^−1^, $F_{\text{ss}}^{\text{PP}}$ = 0. This makes that

6b $$F_{\text{ss}}^{\text{pp}} = F_{\text{ss}}^{\text{pp1}} = nF_{\text{v}} \frac{{k_{\text{L}} }}{{(k_{\text{L}} + k_{\text{AB}}})}$$

Thus the steady state value in the light $F_{\text{ss}}^{\text{PP}}$ of the variable fluorescence associated with primary photochemical quenching is attenuated with respect to the maximal variable fluorescence *nF*_v_ associated with 100 % photochemical reduction of Q_A_. The attenuation factor is determined by the light excitation rate *k*_L_ and the rate of fluorescence (re-) quenching *k*_AB_ in the dark.

**Table:** 

Glossary and description of graphic *F* ^PP^(*t*) parameters (see Fig. 1)	
*F*o	experimental value of *F* ^exp^ in sSP (or SP) at *t* = 0.01 ms; normalization relative to *F*o = 1 has been done
*nF*_v_	approximated by *nF*_v_ = (*F*_m _– 1)/2 in which *F*_m_ is the maximum of *F* ^exp^ at the P-level (see inset)
*k*_L_	slope (ms^−1^) of the initial *F* ^exp^—rise in the 0.01–0.1 time range divided by *nF*_v_
*β*	fraction of *Q*_B_-non reducing RCs in dark-adapted sample; equal to amplitude of extrapolated slow decay component at *t*_0_ (sSP_off_) divided by (1 − exp(−*k*_L_ × 0.25)) to account for the incomplete photoreduction of the fraction *β* at the cessation time (default *t*_0_ = 0.25 ms) of sSP_0.25_
*k*_AB_	decay rate (ms^−1^) of fast decay component; its reciprocal value equals the length of the (green) vertical line (in ms) between the time *t*_0_ (=0.25 ms) of sSP-off and the time at which the SSP-off slope (interrupted black line) intercepts with the decay curve of (red dots)
Approximation and tuning of ‘guess’ parameters to accommodate the matching of *F* ^PP^ at the junction of O–J and J–I phases	
*Ø*	electron trapping efficiency, which is determined by limitation of rate of P^+^ oxidation at PSII donor site by that of radical pair recombination in fraction of Q_b_-nonreducing RCs with single reduced acceptor pair (Vredenberg 2004); actual value, usually in range between 0.1 and 0.5, obtained after varying a tuning factor for matching *F* ^PP^ with *F* ^exp^ at the junction of O–J and J–I phases (see under results)
*k*_2AB_	oxidation rate of double reduced Q_B_-nonreduccing [S0-] RCs in the range between 0.05 and 0.5 ms^−1^ obtained after varying the tuning factor for matching *F* ^PP^ with *F* ^exp^ at the junction of O–J and J–I phases (see under results).

**Fig. 1:**
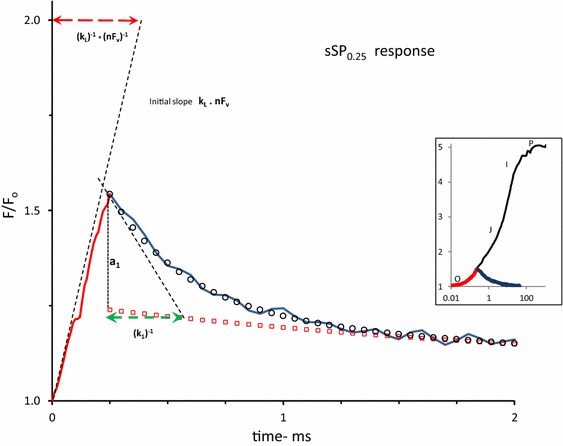
A 2 ms linear time plot of the variable fluorescence *F*(*t*)/*F*_o_ in a *Kalanchoë* leaf during (*red*-*colored line*) and after (*blue*-*colored line*) a short saturating pulse of 250 µs duration (sSP_0.25_) and 3000 µmol photons m^−2^ s^−1^ intensity. The pulse is given at *t* = 0. The insert illustrates the response upon excitation with a 1 s saturating pulse (SP) of the same intensity of 3000 µmol photons m^−2^ s^−1^ on a log time scale and, in *red*, that upon excitation with sSP_0.25_. Upward moving *dashed line* at *t* = 0 is the initial slope of the response at the onset of sSP_0.25_. The downward-directed *dashed line* at the start of the decay at *t* = 0.25 ms is the initial slope of the decay at sSP_off_. *Open black circles* are of the calculated exponential decay $a_{1} .e^{{ - k_{1} .t}}$ which, when supplemented with the residual decay (*open red squared*), simulates the initial *F*(*t*)/*F*_*o*_ decay in the 0.25–1.5 ms time range. Further details are given in Fig. 2 and its legend. The length of the *red*-*colored dashed horizontal line* that connects the point *F*/*F*_o_ = 2 on the *vertical axis* with that of its intercept with the *black*-*colored upward moving line* of the initial slope of the response at *t* = 0, gives the value of the reciprocal of the initial slope. Here the sSP_0.25_-response at *t* = 0 apparently occurs with a reciprocal rate of ~ 350 µs. Similarly, the length of the *green colored horizontal line* from *t* = 0.25 to the intercept of the *slope line* with the (calculated) residual curve (*red squares*) gives a graphical approximation of the reciprocal of the rate constant ((*k*_1_)^−1^) of the fast component

A graphical representation of *F* ^PP^(*t*) (Eq. 6) demands substitution of the values of its constituting parameters (Eqs. 1–5). Relevant data for the estimation of most of these parameters can be derived from (i) the maximum fluorescence level *F*_m_ at the P-level of an SP-induced OJIP induction curve, (ii) the slopes of the initial rise and of the decay components an sSP-induced response, and (iii) the relative amplitudes of the decay components of an sSP-response. The sSP-off decay (Fig. 1) is resolved in three exponential components (not shown) and attributed to the fast and slow plus moderate decay of Q_B_-reducing (open circles) and Q_B_-nonreducing RCs (red dots), respectively. An example is illustrated in Fig. 1. A glossary of the parameters that determine the kinetic profile of the variable fluorescence *F* ^PP^(*t*) associated with primary photochemical quenching is given in the text box (see above).

The analytical solutions (Eqs. 2–4), representing the fluorescence simulation during and after sSP illumination (Fig. 1), illustrate some particular and important aspects of the reaction kinetics of the light–dark reversible de- and re-quenching by Q_A_ in the (photochemical) OJ phase of PSII chlorophyll fluorescence under conditions at which connectivity, donor side-, photoelectrochemical-, and non-photochemical quenching are assumed to be negligible. Firstly, the amplitude of the time-independent equilibrium steady state of the variable fluorescence in the light $F_{\text{ss}}^{\text{PP}}$ is, according to Eq. 6b, dependent on and determined by the light excitation rate *k*_L_ and the rate of fluorescence (re-)quenching *k*_AB_ in the dark. Measurements from which these rates can be determined, like those initiated by short light pulses (Fig. 1) are essential for quantifying the steady state level of the variable fluorescence associated with photochemical quenching. They are for example required for the interpretation of relative changes in the (quasi-)steady state levels of variable fluorescence observed in OJIP induction curves. Secondly, it easily follows from Eqs. 4–6 and recalling that d*F* ^PP2^(*t*)/d*t* = 0 at *t* = 0, that the initial rate (slope) of the variable fluorescence d*F* ^PP^(*t*)/d*t* (=d*F* ^PP1^(*t*)/d*t* = *nF*_v_·*k*_L_) associated with photochemical reduction of *Q*_A_ reduction (de-quenching) is independent of the actual rate *k*_AB_ of its dark re-oxidation (quenching). This means for instance that the initial slope of the photochemical-associated variable fluorescence is unaltered under conditions at which *k*_AB_ = 0. This condition is clearly not fulfilled for the variable fluorescence kinetics in the presence (*k*_AB_ = 0) and absence of the PSII electron transfer inhibitor DCMU. Reasons for the apparent discrepancies and consequences for the validation of quite a number of commonly used trapping models of PSII have been given in Vredenberg and Prasil (2013), but see also Stirbet and Govindjee 2012 for a surveying exposure of pros and cons).

### Thermal JIP phase

The JIP phase has been shown to be composed of two protonophore-sensitive fluorescence components with different kinetic profile (Vredenberg 2011). Systematic analyses of each of these components in low frequency single-turnover flashes (STFs) (Vredenberg et al. 2006, 2007) and in low intensity multi-turnover pulses (SPs) (Vredenberg et al. 2012) have led to a descriptive algorithm, in which the major part of the variable fluorescence during I–J phase in the 0–50 ms time range is given by

7 $$F^{\text{PE}} (t) = 1 + \; nF{\text{v}} \cdot \big[1 - e^{{ - (k_{\text{qbf}} + k_{\text{-qbf}}) \cdot t}} \big] \quad \cdot \,\frac{{k_{\text{qbf}} }}{{k_{\text{qbf}} + k_{\text{-qbf}}}}\left\{1 +\big [1 - q^{\text{dsq}} (t)\big] \quad \cdot \, \big[1 - e^{{- (k_{\text{qbf}} + k_{\text{-qbf}} ) \cdot t}} \big] \cdot\frac{{k_{\text{qbf}}}}{{k_{\text{qbf}} + k_{{\text{-qbf}}} }}\right\}$$

and that of the IP phase in the 50 to 500 ms by

8 $$F^{\text{PE}} (t) = 1 + \; nF{\text{v}} \cdot \big[1 - e^{{ - (k_{\text{qbf}} + k_{\text{-qbf}}) \cdot t}} \big] \, \cdot \,\frac{{k_{\text{qbf}} }}{{k_{\text{qbf}} + k_{\text{-qbf}}}}\left\{1 +\big [1 - q^{\text{dsq}} (t)\big] \,\cdot \, \big[1 - e^{{- (k_{\text{qbf}} + k_{\text{-qbf}} ) \cdot t}} \big] \cdot\frac{{k_{\text{qbf}}}}{{k_{\text{qbf}} + k_{{\text{-qbf}}} }}\right\}$$

A glossary of the additional parameters with which the kinetic profiles of the variable fluorescence *F* ^PE^(*t*) and *F* ^CET^(*t*) can be simulated is given in the text box.

**Table:** 

Glossary and description of graphic *F* ^PE^(*t*)- and *F* ^CET^(*t*) parameters additional to those of *F* ^PP^(*t*)	
*k*_qbf_	rate constant of increase in variable fluorescence during the thermal phase at which the photochemical trapping has reached an equilibrium steady state; it is attributed to the overall rate constant (ms^−1^) of lumenal proton transfer reactions that result in local pH change at the Q_A_–Q_B_ redox side of PSII
*k*_**-**qbf_	rate constant of dark reversion of variable fluorescence induced during the thermal phase; it is attributed to the re-oxidation rate (constant) of RCs with a double-reduced acceptor pair ([PheQ_A_]^2−^)
IP	amplitude of IP phase set equal to *F*_m_^SP ^– [$F_{\text{m}}^{\text{PP}}$ + *F*_m_^PE^]
*k*_IP_	rate constant that determines the increase in *F*_v_ during the I–P phase
*k*_**-IP**_	rate constant determining the major decay component of *F*_v_ in the dark
*N*_IP_	integer (0 < *N*_IP_ < 10) to accommodate delay and steepness of *F*_v_ during I–P phase (*F* ^CET^(*t*))

## Results and interpretation

Figure 1 shows the responses of the variable fluorescence *F*(*t*)/*F*_o_ during and after a short saturating pulse (sSP), in this case of 250 µs duration (sSP_0.25_), and upon excitation with a 1 s saturating pulse (SP) of the same intensity of 3000 µmol photons m^−2^ s^−1^ (insert). The sSP_0.25_-response, plotted on a linear time scale shows at its onset at *t* = 0 (sSP_on_) an initial rise with a reciprocal rate of ~350 µs. This slope, as argued before, is equal to the product of excitation rate *k*_L_ and the (maximum) variable fluorescence *nF*_v_ associated with 100 % reduction of Q_A_ of open RCs. It follows, after application of simple math, that the reciprocal of the slope of the initial rise (in ms) can also be read from the length of the horizontal line that connects the point *F*/*F*_o_ = 2 on the vertical axis with that of its intercept with the (dashed) line of the initial slope. The approximate value of *nF*_v_ can be estimated, as argued before, from *F*_m_ at the P-level of the SP-induced OJIP induction (insert). It gives *nF*_v_ ~ 2. Thus, the excitation rate *k*_L_ for the Kalanchoë leaf illuminated with an (s)SP of 3000 µmol photons m^−2^ s^−1^ intensity is estimated to be *k*_L_ ~ 1.5 ms^−1^. The decay after sSP_off_ is poly-phasic with an initial fast phase and a slow tail extending in the 20 ms time range. Similarly as for the on—rate, the length of the green colored horizontal line from *t* = 0.25 to the intercept of the slope line with the (calculated) residual curve of the tail (red squares) gives a graphical approximation of the reciprocal of the rate constant ((*k*_1_)^−1^) of the fast decay component (open circles). The graph shows, for a pulse duration of 250 µs, that the reciprocal of the initial decay rate (*k*_1_)^−1^ ~ 280 µs.

**Fig. 2:**
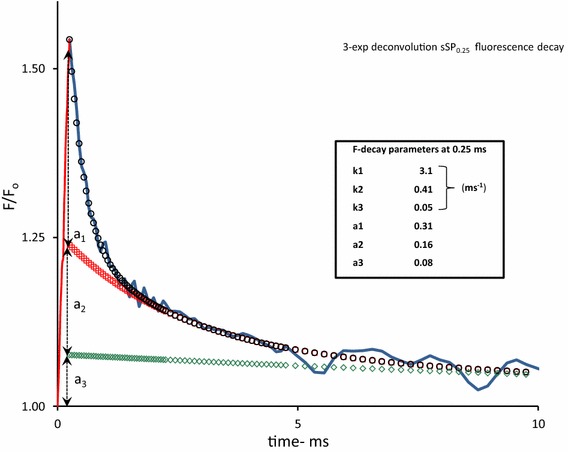
Same experimental sSP_0.25_ response as in Fig. 1 but now plotted on a 10 ms linear time scale. The *F*(*t*) decay after sSP_off_ at *t* = 0.25 is poly-phasic. The symbols in the curve are those of a 3-exponential deconvolution of this decay, with $\frac{F(t)}{{F_{0} }} = \sum\nolimits_{1}^{3} {a_{j} \cdot e^{ - kj \cdot t} }$ The fast, moderate, and slow components, *j* = 1, 2, and 3, are represented by *black open circles*, *red open squares*, and *green open diamonds*, respectively. The *values* of amplitudes (*a*_j_) and rate constants (*k*_j_) of each of the components are given in the *insert box*

Figure 2 shows the 3-exponential deconvolution of the decay on an extended time scale with amplitudes (*a*_i_, *i* = 1–3) and rate constants (*k*_i_, in ms^−1^) of the three components. It illustrates, amongst others, the amply documented heterogeneity of PSII RCs with respect to the re-oxidation rate of their reduced primary quinone electron acceptor ${\text{Q}}_{\text{A}}^{ - }$ by Q_B_, or ${\text{Q}}_{\text{B}}^{ - }$. We presume that the components with *k*_1_ and *k*_2_ are those of RCs in which ${\text{Q}}_{\text{A}}^{ - }$ is re-oxidized by Q_B_ and ${\text{Q}}_{\text{B}}^{ - }$, respectively. This means that under the experimental conditions, owing to this definition and analysis, the rate constant of ${\text{Q}}_{\text{A}}^{ - }$ -re-oxidation *k*_AB_ equals *k*_AB_ = *k*_1_. The slow phase (*k*_3_) of the decay is likely to represent the retarded dark recovery of Q_B_-nonreducing PSII RCs which occurs with rate constant *k*_3_. The amplitude *a*_3_ (Fig. 2) of the extrapolated slow phase of the sSP_off_ decay at *t*_0_ = 0.25 ms (sSP_off_), enables the estimation of the fractional size *β* of Q_B_-nonreducing RCs. The relative size of this fraction with ${\text{Q}}_{\text{A}}^{ - }$ is, according to Eq. 3 and with substitution *k*_L_ ~ 1.25 ms^−1^, equal to 1 – exp(–*k*_L_ × 0.25) = 0.27. This means, with *a*_3_ = 0.08 and *nF*_v_ = 2 (Figs. 1, 2, respectively), that *β* ~ 0.15. Thus the four parameters that define *F* ^PP1^(*t*) (Eqs. 1–4), can be estimated from the kinetic analyses of experimental sSP- and SP-induced responses (Figs. 1, 2).

**Fig. 3:**
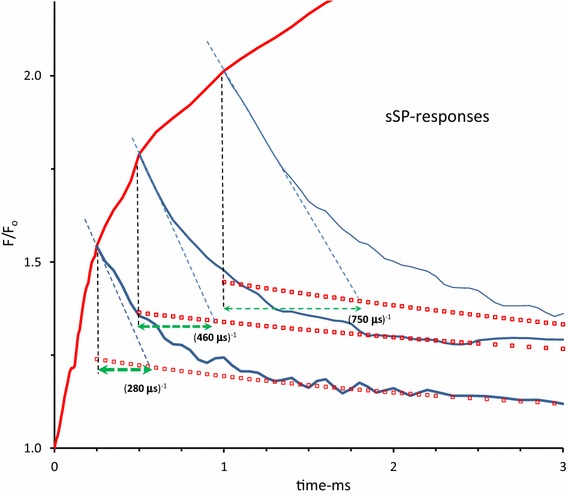
Superposition of the responses of the variable *F*(*t*)/*F*_0_ in a *Kalanchoë* leaf during (*red*-*colored line*) and after (*blue*-*colored*) short saturating pulses of 0.25, 0.5, and 1 ms duration and 3000 µmol photons·m^−2^ s^−1^ intensity in the linear 3 ms time range. Pulses are given at *t* = 0. The ongoing *red* response after *t* = 1 ms is of an 1 s SP curve (see insert Fig. 2). The *red*-*colored squares* mark the decay of summed moderate and slow phase and the *green colored dashed lines* the approximate reciprocal of the rate constant of the fast decay component of the respective sSP-responses (see further Fig. 2). Mark, in particular, the increase length of these *horizontal lines* with increase in duration of the pulses

**Fig. 4:**
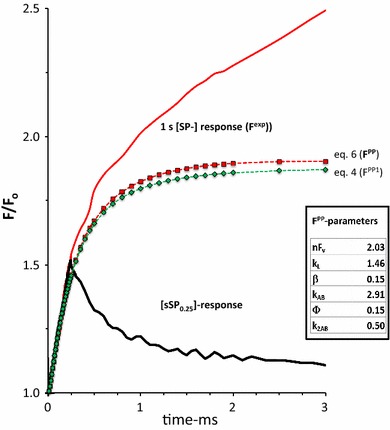
The 3 ms linear time plot of the variable fluorescence response in a *Kalanchoë* leaf upon a 0.25 ms sSP_0.25_ (*black*-*colored*) and a 1 s SP (*red*-*colored*) both of 3000 µmol photons·m^−2^ s^−1^ intensity. The two pulses are given sequentially at an intermediate dark period of a few seconds. The curves with *green diamonds* and *red squares* are the *graphic plots* of the calculated variable fluorescence associated with the release of primary photochemical quenching without (*F* ^PP1^) and with (*F* ^PP^) supplemental quenching release associated with double reduction of Q_B_-nonreducing RCs, respectively. *F* ^PP1^ and *F* ^PP^ are calculated using Eqs. 4 and 6 with substitution of the parameter values estimated from the kinetic- and steady state analyses illustrated in Figs. 1 and 2, except *φ* and *k*_2AB_, and are given in the table in the insert

Figure 3 shows a reproduction and decay analysis of three subsequent excitations with short saturating pulses of 0.25, 0.5, and 1 ms duration, plotted on top of each other in one figure. The increase in length of the horizontal line connecting the vertical line at sSP_off_ and the intercept of the slope at light off with the extrapolated curve of the moderate plus slow phase, indicates an increase in the rate constant *k*_AB_ with the length of the light (pulse) period in the 0.01–1 ms time range. This is presumed to be due to the light-driven pH shift of the ${\text{Q}}_{\text{A}}^{ - }$Q_B _↔ Q_A_ ${\text{Q}}_{\text{B}}^{ - }$ redox equilibrium accompanying progressing H^+^ uptake at the Q_r_ -site of the cytb_6_f complex (Vredenberg and Prasil 2009, 2013). Figure 4 shows, on a linear 3 ms time scale the graphic plot of *F* ^PP1^(*t*) resulting from application of Eq. 4. The supplementary contribution of fluorescence de-quenching associated with double-hit electron trapping in Q_B_-nonreducing RCs [Eq. 5, *F* ^PP2^(*t*)], is seen by comparing the graphs of *F* ^PP^ (Eq. 6) and *F* ^PP1^ (Eq. 4) in Fig. 4. The electron trapping efficiency *Φ* in the second excitation (hit) has been set at *Φ* = 0.15. This gives a closest fit of *F* ^PP^ + *F* ^PE^ with *F* ^exp^ in the 1–5 ms time range, as will be discussed below. The steady state $F_{\text{ss}}^{\text{PP}}$ ~ 0.9 of *F* ^PP^(*t*) (with reference to *F*_o_ = 1) is reached after about 10 ms (not shown). This equilibrium state is determined by the fraction *q* ^dsq^ of centers that has become photochemically closed in the light. It follows (Eq. 2) that, at the intensity used, this fraction amounts 0.85 × 1.5/4.3 + 0.15 = 0.45.

**Fig. 5:**
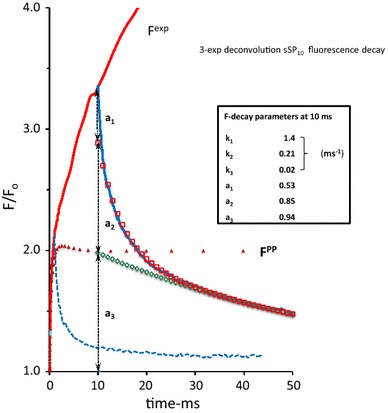
The 50 ms linear time plot of the variable fluorescence response (*F* ^exp^(*t*)) in a *Kalanchoë* leaf upon a 10 ms short saturating pulse sSP_10_ (*blue*-*colored*) and a 1 s SP (*red*-*colored*), both of 3000 µmol photons m^−2^ s^−1^ intensity. The two pulses are given sequentially at an intermediate *dark period* of a few seconds; the 10 ms rising part of the sSP_10_ response coincides with that of the SP response. The *green open diamonds* and *red open squares* are of the slow (*k*_3_) and moderate(*k*_2_) component, respectively of the 3-exponential deconvolution of the sSP_10_ decay curve. Values of amplitudes (*a*_j_) and rate constants (*k*_j_) of each of the components are given in the insert. The *blue dashed line* is, for comparison and reproduced from Fig. 1, the decay of the sSP_0.25_ response. The red colored triangles are of the variable fluorescence curve *F* ^PP^ (*t*), reproduced from Fig. 4. Note the substantial increase of, in particular the amplitude of the slow (*a*_3_) and moderate (*a*_2_) components of the *F*(*t*) dark decay associated with the *F*(*t*) rise in the 10 s time domain

Figure 5 shows, for the same leaf as in Figs. 1, 2, 3, 4, the variable fluorescence *F*(*t*)/*F*_o_ in the (linear) time range of 20 ms during the 1 s saturating pulse of 3000 µmol photons m^−2^ s^−1^ and in the 40 ms dark period after a short 10 ms saturating pulse (sSP_10_). The decay after sSP_10_ is resolved, after exponential deconvolution, into three components with amplitudes (*a*_i_, *i* = 1–3) and rate constants (*k*_i_, in ms^−1^). The values of these F-decay parameters are given in the inset table. Comparison of the decay patterns after sSPs of 1 and 10 ms duration shows that that the fluorescence dark kinetics after short pulse excitation substantially changes with the length of the pulse. Most pronounced is the increase in the slow (*a*_3_) decay component that apparently has accumulated during the extension of the pulse period from 1 to 10 ms. This would suggest, in terms of TSTM, a stimulated accumulation and photochemical reduction of reduced Q_B_-nonreducing RCs in the light period and dark re-oxidation with rate constant *k*_3_ (~0.02 ms^−1^). It is further noticeable that the rate constants of the decay components have continued to decrease during the extended sSP duration.

**Fig. 6:**
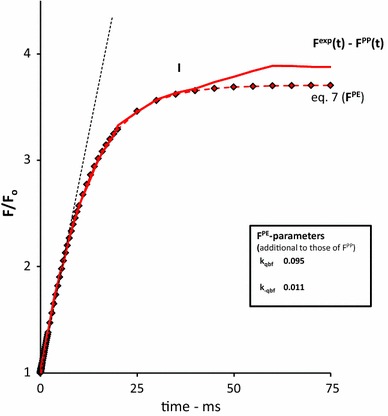
The 75 ms linear time plot of the variable fluorescence response (*F* ^exp^(*t*) − *F* ^PP^(*t*)), complementary to the rise exclusively caused by the release of primary photochemical quenching, upon a 1 s saturating pulse SP (*red line*) of 3000 µmol photons m^−2^ s^−1^ intensity in a *Kalanchoë* leaf. The curve is obtained after subtraction *F* ^PP^(*t*) (see Fig. 4, 5) from *F* ^exp^(*t*) (see insert Fig. 2). The curve with red diamonds is the graphic plot of Eq. 7, attributed to *F* ^PE^(*t*), with substitution of the parameter values estimated for *F* ^PP^(*t*) (see insert Fig. 4) and supplemented with those estimated for *k*_qbf_ and *k*_−qbf_, given in the insert table, to obtain the best fit with the experimental curve in the 0 to 30 ms time domain, after ‘correction’ for *F* ^PP^(*t*)

Figure 6 (red colored line) shows, on a linear 75 ms time scale, the plot of *F* ^exp^(*t*) after subtracting *F* ^PP^(*t*). It shows a bi-phasic increase in fluorescence with an inflection point (at level I) at a time around 30 ms at which a second rise becomes apparent. It is obvious from the slope of the response (dotted line), as compared to that of the initial rise (Fig. 2), that the rate constant of the transfer or process that is responsible for the light-driven increase in variable fluorescence is substantially lower than that of the photochemical conversion at *t* = 0 (Fig. 1). The red colored diamonds and dotted line in Fig. 6 are those calculated with Eq. 7 for the variable fluorescence *F* ^PE^(*t*) attributed to photo-electrochemical transfer of RCs into the Q_B_-nonreducing form and the sequential trapping, with attenuated efficiency *Ø*, of a second electron in subsequent hits (excitations). A close matching between experimental (*F* ^exp^ − *F* ^PP^) and simulation (*F* ^PE^) curves is obtained with rate constants for the forward light (*k*_qbf_) and reversal back reactions (*k*_−qbf_) of about 0.1 and 0.01 ms^−1^, respectively. The steady state of the light-driven photo-electrochemical thermal (J–I) phase is *F*_ss_^PE^(*t*) ~ 2.5.

**Fig. 7:**
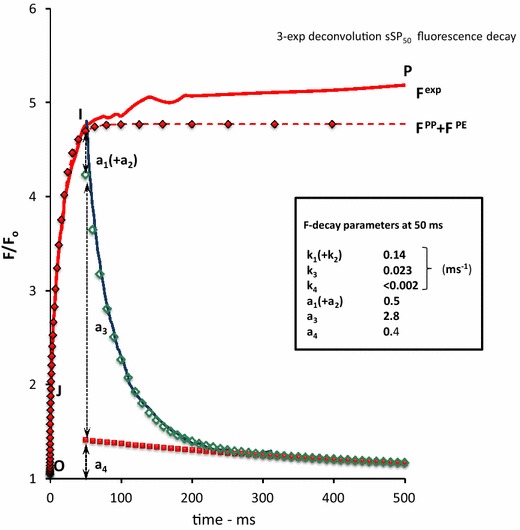
The 500 ms linear time plot of the variable fluorescence response (*F* ^exp^(*t*)) in a *Kalanchoë* leaf upon a 50 ms sSP_50_ (*blue*-*colored*) and a 1 s SP (*red*-*colored*), both of 3000 µmol photons m^−2^ s^−1^ intensity. The two pulses are given sequentially at an intermediate dark period of a few seconds; the 50 ms rising part of the sSP_50_ response coincides with that of the SP response. The *green* *open open diamonds* and *closed red squares* are of the intermediate (*k*_2_) and slow (*k*_3_) component, respectively of the 3-exponential deconvolution of the sSP50 decay curve. Values of amplitudes (*a*_j_) and rate constants (*k*_j_) of each of the components are given in the insert. The *red colored diamonds* are of the variable fluorescence curve *F* ^PP^(*t*) + *F* ^PE^(*t*), after summation of the respective curves from Fig. 4 (*F* ^PP^) and Fig. 6 (*F* ^PE^), respectively. Note the appearance of the ultra-slow decay component ((*k*_3_)^−1^ ~ 0.5 s) in the decay at 50 ms and (ii) approx. equal size of the rate constant k2 in the sSP_50_ decay and k3 in the sSP_10_ decay (see Fig. 5)

Figure 7 shows, on a 500 ms linear time scale, the variable fluorescence of the same leaf upon a 1 s SP and during and after a 50 ms saturating pulse (sSP_50_). The closed red diamonds are those obtained after summation of the calculated *F* ^PP^(*t*) and *F* ^PE^(*t*) curves of Figs. 4 and 6. They show the closes fit with *F* ^exp^ in the 0–50 ms time range and steady state equilibrium above that range. The results of the 3-exponential deconvolution of the decay after sSP_50_ are summarized in the table in the insert and illustrated with symbols in the decay curve. The major (75 %) contribution of the decay is of a component that recovers with a rate constant *k*_3_ (~0.02 ms^−1^). A component with about the same rate constant was apparent in the decay after sSP_10_, as illustrated in Fig. 5. The similarity between the increase in size of this component [from ~0.9 at 10 ms (Fig. 5) to ~2.8 at 50 ms (Fig. 7)] with that of the fluorescence response suggests that the variable fluorescence in the light in the time domain of 1–50 ms is under control of this component. The reasonable correspondence between the value of the rate constant of the decay after sSP_50_ (Fig. 7) and that of the reversal reaction (*k*_−qbf_) introduced for simulation (Eq. 7) of the variable fluorescence associated with photo-electrochemical quenching *F* ^PE^(*t*) is in agreement with this hypothesis.

**Fig. 8:**
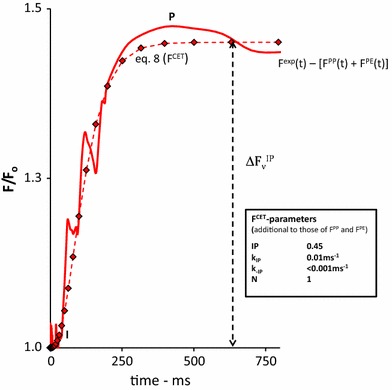
The 750 ms linear time plot of the variable fluorescence response $\left( {F^{\exp } (t) - \left[ {F^{\text{pp}} \left( t \right) + F^{\text{pp}} \left( t \right)} \right]} \right)$, complementary to the fluorescence rise caused by *F* ^PP^(*t*) and *F* ^PE^(t), upon a 1 s saturating pulse SP (*red line*) of 3000 µmol photons m^−2^ s^−1^ intensity in a *Kalanchoë* leaf. The curve is obtained after subtraction the sum of *F* ^PP^(*t*) and *F* ^PE^(*t*) from *F* ^exp^(*t*) (see Fig. 7). The curve with *red diamonds* is the graphic plot of Eq. 8 attributed to *F* ^PE^(*t*), with substitution of the parameter values estimated for *F* ^PP^(*t*) (see insert Fig. 4) and supplemented with those estimated for *k*_qbf_ and *k*_−qbf_, given in the insert table, to obtain the best fit with the experimental curve in the 0 to 30 ms time domain, after ‘correction’ for *F* ^PP^(*t*)

The red solid curve in Fig. 8 is the linear time plot of *F* ^exp^(*t*) after subtracting the variable fluorescence associated with release of photochemical [*F* ^PP^(*t*)] and photo-electrochemical quenching [*F* ^PE^(*t*)]. It shows an approx. 0.45 increase ($\Delta F_{\text{v}}^{\text{IP}}$) in variable fluorescence in the light toward the final P level at the maximum fluorescence *F*_m_. The response shows a delay of approx. 30 ms and reaches its equilibrium state after about 300 ms. The red diamonds are the fluorescence values of the simulation curve *F* ^CET^(*t*) using Eq. 8 and substituting parameter values listed in the insert of the figure.

**Fig. 9:**
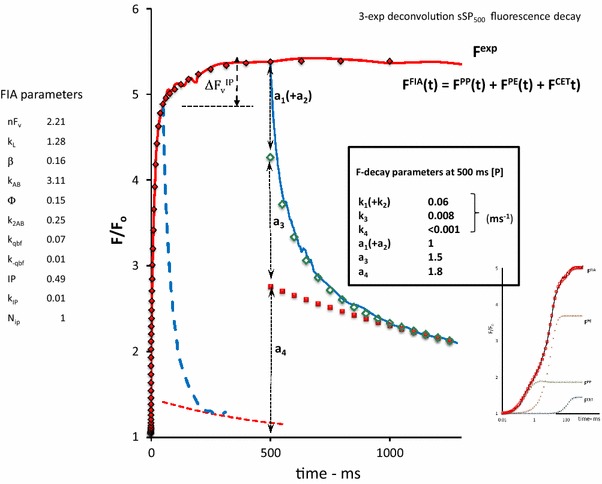
The 1300 ms linear time plot of the variable fluorescence response (*F* ^exp^(*t*)) in a *Kalanchoë* leaf upon a 500 ms (s)SP_500_ (*blue*-*colored*) and a 1 s SP (*red*-*colored*), both of 3000 µmol photons m^−2^ s^−1^ intensity. The two pulses are given sequentially at an intermediate dark interval of a few seconds; the rising part of the (s) SP_500_ response coincides with that of the SP response. The green diamonds and *red squares* are of the intermediate (*k*_3_) and slow (*k*_4_) components, respectively of the 3-exponential deconvolution of the sSP_500_ decay curve. Amplitudes (*a*_j_) and rate constants (*k*_j_) are given in the insert. The *blue dashed line* is the decay of the sSP_50_ response and the *red dashed curve* is of its slow decay component (both reproduced from Fig. 7). The *red diamonds* are of the FIA-simulation curve *F* ^FIA^(*t*) = *F* ^PP^(*t*) + *F* ^PE^(*t*) + *F* ^CET^(*t*) resulting after summation of the respective curves from Fig. 4 (*F* ^PP^), Fig. 6 (*F* ^PE^) and Fig. 8 (*F* ^CET^), respectively. The parameters of the constituting components of *F* ^FIA^(*t*) (Eqs. 6–9) are given in the left hand panel. The *bottom-right insert* gives the results plotted on a log time scale. *Note* (i) the nice similarity between *F* ^exp^(*t*) and *F* ^FIA^(*t*) and (ii) the substantial increase in the contribution of the ultra-slow decay component ((*k*_3_)^−1^ ~ 1 s) that has occurred during the 50–500 ms light period (IP phase) in which *F* ^exp^ has increased with a comparably smaller amount $\Delta F_{\text{v}}^{\text{IP}}$

The variable fluorescence response upon a 1.3 s saturating pulse of 3000 µmol photons m^−2^ s^−1^ and of the fluorescence dark decay after a similar pulse of 500 ms duration (sSP_500_) is illustrated in Fig. 9. The characteristic parameters of the three exponential decay components are given in the insert. The decay after a 50 ms short pulse (sSP_50_), reproduced from Fig. 7, is drawn for comparative reasons. A prominent growth, concurrent with the increase in variable fluorescence in the light, is seen in the size of the slow decay component (*a*_3_) with a reciprocal rate of approx. (10 s)^−1^ which has occurred during the final part (IP) of the thermal phase in the 50–500 ms time range. The summed values *F* ^PP^(*t*) + *F* ^PE^(*t*) + F^CET^(*t*) which constitute the simulation *F* ^FIA^(*t*) of the experimental variable fluorescence induction curve *F* ^exp^(*t*) are also shown as red colored diamonds. The FIA parameters are listed in the left hand table. The same results for *F* ^exp^ and *F* ^FIA^, but plotted on a commonly used log-time scale and complemented with the constituting components of *F* ^FIA^ are illustrated in the bottom-right hand insert.

**Fig. 10:**
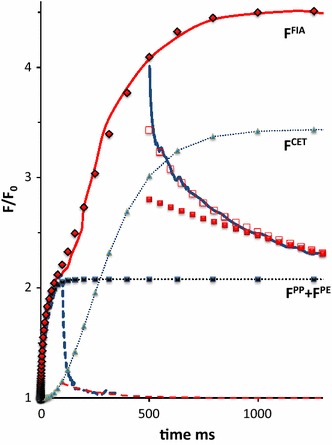
Linear time plot, similar as in Fig. 9, except for ten-fold attenuation of pulse intensity and plant (leaf) species, of *F* ^exp^(*t*) in a *Arum italliensis* leaf upon a 500 ms (s) SP_500_ and a 1 s SP, both of 300 µmol photons m^−2^ s^−1^ intensity. Here the *blue dashed line* is the decay of the sSP_100_ response. *Meaning of symbols* *and labeled curves* is the same as in Fig. 9

Figure 10 shows the same experiment as Fig. 9 but done at a tenfold lower intensity of the actinic light pulse and in a leaf of a different plant species. The results on the light response at low(er) intensities are in agreement with those of similar experiments in many other plant species (Strasser et al. 1995; Lazár 2006; Schansker et al. 2006; Vredenberg 2011) and demonstrate (i) an apparent increase $\Delta F_{\text{v}}^{\text{IP}}$ of *F* ^CET^, a much lower OI phase (*F* ^PP^ + *F* ^PE^) and, (iii) nearly the same *F*_m_ as compared to values at a tenfold higher intensity shown in Fig. 9. However, the slow (*k*_4_) component of the decay is as large as observed at the higher intensity in Fig. 9. This observation, as will be discussed later, hints to the conclusion that the de-quenching process responsible for the IP phase is mechanistically different from those of the O–J–I phase.

**Fig. 11:**
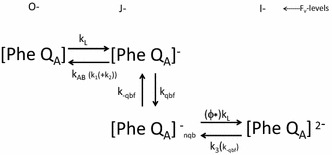
Schematic scheme representing the reactions that cause light-induced variable fluorescence associated with *F* ^PP^ and *F* ^PE^ in the OJI phase. Rate constants characteristic for* forward* and* backward *reactions are indicated. The upper reaction scheme of the photochemical O–J phase and associated with *F* ^PP^. The* vertical* and bottom scheme is of the J–I phase. Further explanations are in the text

Figure 11 gives a reaction scheme for the light-induced variable fluorescence associated with *F* ^PP^ and *F* ^PE^, in which the estimated rate constants which are characteristic for forward and backward reactions are indicated. The upper line represents the reaction scheme of the photochemical O–J phase which is described by *F* ^PP^. The vertical reaction scheme in the middle represents the photo-electrochemical transfer reaction of ‘normal’ RCs with ${\text{Q}}_{\text{A}}^{ - }$ and *k*_AB_ > 0 into Q_B_-nonreducing RC with *k*_AB_ = 0 (indicated by the subscript _nqb_). The bottom scheme is of the reversible photochemical reduction of Q_B_-nonreducing RCs in which a 2nd electron is trapped. These latter two sequential reactions are representative for *F* ^PE^ involved in the I–J phase.

**Fig. 12:**
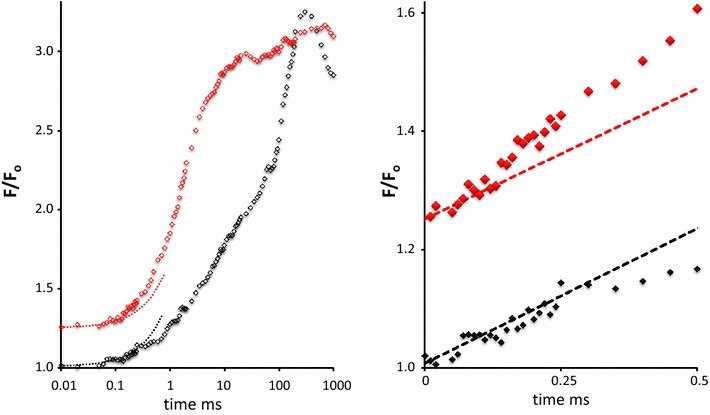
Left hand panel. Variable fluorescence in aqueous suspension of *Nannochloropsis* upon 1 s SP (500 μmol photons m^−2^s^−1^) in absence (*blacks*) and presence (*reds*) of 1 μM DCMU). The herbicide was added in strict darkness. Data are average of 12 experiments with three samples. *Right hand panel*: Same data in the 0 to 0.5 ms time range as in *left hand panel*, but plotted on a linear time scale. It shows the following properties: *F*o in the presence of DCMU has increased to 1.25 with unaltered *F*_m_ ~ 3*F*_o_. Initial rate of fluorescence increase at onset of SP is insensitive to DCMU (*dashed lines* in right hand panel). Initial fluorescence increase in the presence of DCMU is sigmoidal

The left hand part of Fig. 12 shows the amply documented effect of DCMU addition on the induction pattern of the variable chlorophyll fluorescence, measured here in an aqueous suspension of *Nannochloropsis* and plotted on a logarithmic time scale. The initial response in the (linear) 0.5 ms time domain is reproduced for the same experiment in the right hand part. The results in the presence of DCMU illustrate (i) the initial rate of variable fluorescence is not affected, and (ii) an increasing rate of the *F*_v_ rise after a delay of ~100 µs. The latter observation makes the rise sigmoidal.

## Discussion

The poly-phasic so-called OJIPSMT time pattern of variable chlorophyll fluorescence in algae and intact leaves is generally considered as a valuable source of information on the primary and secondary photosynthetic processes that are involved in bioenergy conversion and biomass production (Kautsky and Hirsch 1931; van Kooten and Snel 1990; Papageorgiou and Govindjee 2004; Suggett et al. 2010; Kalaji et al. 2012). The large amount of experimental fluorescence data, collected from experiments with algae, leaves, chloroplasts, and fragments thereof, has not led so far to a covering interpretation model that has received general acceptance (Stirbet and Govindjee 2012). A likely and probably major reason is a fundamental disagreement on the interpretation of the maximal fluorescence *F*_m_. *F*_m_ is measured at the P-level of the OJIP rise, starting at O from *F*_o_, after a light period variable between 200 and 700 ms, depending on light intensity (Figs. 8, 9, 10). *F*_m_ is reached at much shorter times in the presence of DCMU (see for instance Fig. 12). *F*_m_ is considered) to be exclusively associated with the complete release of photochemical quenching. Reference is then made to the classic paper of Duysens and Sweers (1963) in which convincing evidence has been presented that photochemical reduction of Q_A_, which acts as an antenna fluorescence quencher, leads to RC closure and consequently, like in photosynthetic bacteria for the photochemical oxidation of the reaction center (bacterio-)chlorophyll (Vredenberg and Duysens 1963), to an increase in chlorophyll fluorescence yield. However, application of one of the rules of logics does not allow the reverse conclusion that an increase in fluorescence yield is only associated with the photoreduction of a quencher. Thus the assumption that in algae and leaves the maximal variable fluorescence *F*_m_ due to closure the RCs of PSII is exclusively associated with full release of photochemical quenching by Q_A_ is not necessarily correct and requires substantiation and validation. The results of experiments on the light-on and light-off responses of *F*_v_ with sSPs in the range between 250 and 1000 ms and the kinetic analyses thereof (Figs. 1, 2, 3, 4) illustrate unequivocally that the maximal variable fluorescence nF_v_ (~2 for Kalanchoë) associated with complete reduction of Q_A_ (Fig. 4) is less than the maximal variable fluorescence (~4 for Kalanchoë) associated with *F*_m_ ~ 5 (insert Fig. 2). This conclusion is in firm agreement with that obtained with other approaches and species, amongst which estimates that the maximal variable fluorescence *F*_m_^STF^ in (µs-) saturating single-turnover flashes is by 25–50 % less than the maximal variable fluorescence *F*_m_ in (>300 ms-) multi turnover light pulses, documented for a large variety of algae and plant chloroplasts (Samson and Bruce 1996; Koblizek et al. 2001; Vredenberg et al. 2007; Vredenberg and Prasil 2009; Klughammer and Schreiber 2015).

I will now turn to the analyses of light and dark kinetics of variable fluorescence in the subsequent photochemical (O–J) and thermal phases (J–I and I–P) of the pulse-induced variable fluorescence in intact leaves. Till now, simulated curves of variable chlorophyll fluorescence *F* ^FIA^(*t*) were obtained by substituting proper values of the constituting parameters in the equations of its photochemical [Eqs. 1–6, *F* ^PP^(*t*)] and photo-electrochemical components (Eqs. 7–8 for *F* ^PE^(*t*) and *F* ^CET^(*t*),respectively). The estimates of the parameters (i) guaranteed the closest fit of *F* ^FIA^(*t*) (=*F* ^PP^(*t*) + *F* ^PE^(*t*) + *F* ^CET^(*t*)) with the experimental curve *F* ^exp^(*t*) and (ii) were, for each, within the range of values estimated or concluded in experiments with (sub-) cellular or organellar preparations under comparable conditions. This methodology of our system analysis approach however has been judged as a weakness and deprivation (Stirbet and Govindjee 2012). If it were, this imperfection is overcome for a great deal with the application of sub-saturating pulses (sSPs).

### Photochemical O–J phase; *F*^PP^(*t*)

There are instrumental limitations for applying STFs in fluorescence studies with intact leaves because of the inability of existing LED assemblies to reach fluorescence saturation within the 10 µs time range. This excludes the possibility of estimating the actual value of *nF*_v_ with *µ*s-STFs. The application of sSPs in an extended time range and the monitoring of the light-on and light-off variable fluorescence kinetics at an adjustable time resolution above 10 µs has dissolved this limitation. But there are more advantages of using these short duration pulses. So far the simulation and fitting of experimental OJIP (SMT) curves were done starting from the set of equations that describe the reaction kinetics of photochemical quenching of fluorescence (i.e., Eqs. 1–6) supplemented with those for fitting the JIP phase (i.e., Eqs. 7–8) of the OJIP induction curve. These form, except for some slight modifications, the framework of the fluorescence induction algorithm FIA that has been introduced in earlier reports.

The data of Figs. 1, 2, 3, 4 illustrate that implementation of tools enabling high time resolution of fluorescence (*F*_v_) responses and mathematical handling of underlying reaction kinetics serves the approximation and/or estimation of the actual values of the determinant parameters of *F* ^PP1^(*t*) in a leaf under the measuring conditions: (1) the slope of the initial *F*_v_ rise at light on (Fig. 2) equals the product of light excitation rate (*k*_L_) and maximal variable fluorescence (*nF*_v_) associated with release in photochemical quenching and (2) the slope of *F*_v_ at light off (Figs. 2, 3) and the decay pattern in the dark (Fig. 1) give estimates of the actual values of the rate of oxidation of ${\text{Q}}_{\text{A}}^{ - }$ by Q_B_ (*k*_AB_ = *k*_1_ in Fig. 1) and presumably by ${\text{Q}}_{\text{B}}^{ - }$ (*k*_2_ in Fig. 2) and of the fraction *β* of Q_B_-nonreducing RCs. With actual data read from *F*_m_ (insert Fig. 2), the on- and off-slopes of sSP-induced *F*_v_ responses (Fig. 2) and from the 3-exponential decay analysis (Fig. 1), *F* ^PP1^(*t*) can be estimated using Eq. 4. A first matching of *F* ^PP^(*t*) with the initial phase of *F* ^exp^(*t*) usually is done by manually varying the magnitude of *k*_AB_ with a small percentage. For example, an increase in *k*_AB_ will cause (Eq. 1) a downward movement of the *F* ^PP^(*t*) without affecting the initial slope, as outlined in an earlier section. The choice of using *k*_AB_ as a fine tuner for the matching is not surprising in view of the fact that, as illustrated in Fig. 3, this rate constant of dark oxidation of ${\text{Q}}_{\text{A}}^{ - }$ decreases during the approx. 1 ms rise period of *F* ^PP^ (*t*). This means that the actual average value of *k*_AB_ is less than the one that is estimated as a rule from the dark decay at 250 µs (Fig. 2). Moreover, and in order to simplify the calculations, the fluorescence decay attributed to oxidation of ${\text{Q}}_{\text{A}}^{ - }$ has been approximated by a single exponential. This means that the *F*_v_ decay, after correction for the slow decay with rate constant below ~0.05 ms^−1^, i.e., with amplitude(*a*_1_ + *a*_2_) (Fig. 1) is simulated with one reciprocal rate constant that equals the time *t* at which *F*_v_ has decreased toward a value ~0.37 × (*a*_1_ + *a*_2_). In the experiment of Fig. 1 this would have given (not shown) a value *k*_AB_ ~ 1.8 ms^−1^. A next refined matching of *F* ^PP^(*t*) with *F* ^exp^(*t*) (Fig. 4) is done by varying the magnitude of electron trapping efficiency *Ø* in fraction *β* of Q_b_-nonreducing RCs with single-reduced acceptor pair. It allows a fine tuning of the rise of *F* ^PP2^(*t*) and serves a matching of *F* ^PP^ with *F* ^exp^ at the junction of O–J and J–I phases in the 0.5–1.5 ms time range.

Thus the variable fluorescence *F* ^PP^(*t*) associated with release of photochemical quenching can be estimated with reasonable precision and accuracy. The example displayed for a Kalanchoë leaf (Fig. 4) but qualitatively representative for leaves of other plant species, illustrates that, at the intensity used, the release associated with photochemical quenching reaches an equilibrium state at $F_{\text{ss}}^{\text{PP}}$ (=*F*/*F*_o_ − 1) ~0.9 after about 1.5 ms, at which (e.g., Eq. 6b) ~45 % of Q_A_ has become reduced. The figure and Eq. 6b predict a strong dependence of $F_{\text{ss}}^{\text{PP}}$ on *k*_L_ (actinic light intensity) and on *k*_AB_. A tenfold attenuation in light intensity will lower $F_{\text{ss}}^{\text{PP}}$ from 0.9 to ~0.3, and a treatment causing *k*_AB_ = 0 (for instance addition of DCMU) is predicted to result in its rise toward *nF*_v_ (~2). The latter is in conflict with experimental data (see for instance Fig. 12), which show that in the presence of DCMU *F* ^exp^(*t*) reaches a value $F_{\text{DCMU}}^{ \exp } \sim F_{\text{m}}$ (~5). This seeming discrepancy has received ample attention in earlier reports (for survey, see Vredenberg and Prasil 2013). The variable fluorescence not associated with primary photochemical quenching and referred to as being associated with the thermal phase is obtained by subtracting *F* ^PP^(*t*) from *F* ^exp^(*t*).

### Thermal J–I–P phase, J–I component, *F*^PE^(*t*)

The first part of the thermal phase covering the time domain between ~2 and 50 ms, denoted as the J–I phase, has been interpreted in most models to be caused by processes associated with PQ reduction. The identity of these processes is still under debate (for a survey see Stirbet and Govindjee 2012). An interesting observation has been reported which showed, at an unaltered *F*_m_, a largely suppressed J–I and a stimulated I–P phase in the OJIP induction of etiolated wheat leaves after a greening period of 24 h (Dinc et al. 2012). This effect may hint to a relation of the occurrence of the involved reaction with the assemblage of the photosynthetic machinery. The application of sSPs with a duration that covers the J–I component of the thermal phase in the time domain of tens of ms (Figs. 5, 7), has shown (i) a substantially lower rate for *F*_v_ in the light, (ii) an approx. twofold decrease of *k*_1_ and *k*_2_ in the dark, attributed to (re-) quenching by Q_A_ and contributing ~60 % (=100 × (*a*_1_ + *a*_2_)/(*a*_1_ + *a*_2_ + *a*_3_)) of the total *F*_v_ at *t* = 10 ms (Fig. 5), and (iii) a growth in the contribution of the slow *k*_3_-component in the dark decay from ~14 % at 0.25 ms (Fig. 1) to ~40 and 75 % at 10 and 50 ms, respectively (Figs. 5, 7). The fact that the *F*_v_ increase during the J–I phase in the light is accompanied by an increase in the slow *k*_3_-decay phase of comparable size leads to the conclusion that the responsible light-driven process of the J–I phase reverses in the dark with a rate constant of the order of 0.02 ms^−1^. The process apparently is active under conditions at which Q_A_ is photochemically reduced in more than 50 % of the fraction of the RCs. An increase in this fraction during the J–I phase is obvious from the decrease in the amplitude (*a*_1_ + *a*_2_) attributed to photochemical reduction of Q_A_. The simultaneous appearance of an *F*_v_ component (*a*_3_) which relaxes (*k*_3_) in the dark with an approx. 20-fold slower rate, and the observation that the amplitude *a*_3_ of this component at the end of the J–I phase (i.e., at ~50 ms (Fig. 7)) exceeds $F_{\text{m}}^{\text{PP}}$ (=*nF*_v_) give support for the hypothesis (Vredenberg et al. 2006; Vredenberg and Prasil 2009) that the component results from variable fluorescence *F* ^PE^(*t*)associated with RC closure caused by electron trapping in (semi-closed) RCs with ${\text{Q}}_{\text{A}}^{ - }$, after their photo-electrochemical conversion into the Q_B_-nonreducing form. The simulation of *F* ^exp^(*t*), after subtraction of *F* ^PP^(*t*), with *F* ^PE^(*t*) using the equation (Eq. 7) that represents the reaction kinetics of this double-hit trapping mechanism gives additional support for the hypothesis (Fig. 6). The simulation was done using the parameters estimated for *F* ^PP^(*t*) (see Fig. 4) complemented with an excitation *k*_qbf_ ~ 0.1 (~0.07 *k*_L_) and relaxation rate *k*_−qbf_ ~ 0.01 (both in ms^−1^). The latter compares reasonably with the estimated relaxation rate *k*_3_ of the major component of the *F*_v_ decay during the J–I phase. The attenuated excitation rate *k*_qbf_ of *F* ^PE^(*t*) as compared to that of *F* ^PP^(*t*) has been ascribed to the rate of the pH change at the Q_A_ − Q_B_ reducing site which results from a proton flux, competitive with the larger flux from non-specific H^+^ sources, toward the light-driven H^+^ uptake at the Q_B_ site that occurs at the excitation rate *k*_L_ (Vredenberg 2011). The pH change at the Q_A_ − Q_B_ reducing site is reflected by the decrease in the rate of the initial dark decay *k*_1_ and *k*_2_, ascribed to the ${\text{Q}}_{\text{A}}^{ - }$ re-oxidation rate *k*_AB_ with increasing duration of light pulses (Figs. 2, 5, 7, 9).

### Thermal J–I–P phase, I–P part, *F*^CET^(*t*)

There is as yet no consensus on the origin of the IP phase, except for the conclusion that its appearance in the OJIP induction curve requires the activity of PSI (Bulychev and Vredenberg 2001; Schansker et al. 2005; Joly and Carpentier 2009; Ceppi et al. 2011; Vredenberg 2011). Short saturating pulses (sSPs), with a duration that covers the I–P component in the time domain of hundreds of ms, give interesting information on the process that is driving *F*_v_ during the I–P phase (Figs. 7, 8, 9). At the intensity used (3000 µmol photons.m^−2^ s^−1^), *F*_v_ has increased during the I–P phase from a value ~4.8 at I toward ~5.4 at P in the time span between 50 and 500 ms. The light processes at level I showed, upon termination at *t* = 50 ms, a poly phasic dark decay of *F*_v_ (Fig. 7). The major component (*a*_3_) reverses with a reciprocal rate constant of ~50 ms and is followed by a component with amplitude *a*_4_ ~ 0.4 and a reciprocal rate exceeding 500 ms. This pattern is distinctly different from that at the P-level (*F*_m_) at 500 ms at which the major component has decreased and the slow one has raised its amplitude toward *a*_4_ = 1.8 (Fig. 9). The increment of this dark decay component with reciprocal rate of about 1 s is disproportional with the relatively small increase in variable fluorescence ($\Delta F_{\text{v}}^{\text{IP}}$) during the I–P phase (Fig. 9). This phenomenon sets a constraint to the properties of the process that is responsible for the *F*_v_ increase during the I–P phase, in particular in relation to those that associated with *F* ^PP^ and *F* ^PE^. The *F*_v_ increase during the I–P phase has been termed *F* ^CET^, and has been attributed to a photo-electrical stimulation of the fluorescence yield by cyclic electron transport CET powered by PS1 Vredenberg (2008b, 2011). *F* ^CET^(*t*) has been derived (Fig. 8) by estimating the best fit for the residual curve obtained after subtracting the sum of *F* ^PP^(*t*) and *F* ^PE^(*t*) from *F* ^exp^(*t*) using Eq. 8. This equation has been discussed to account for the (variability in) sigmoidicity and steepness of the I–P curve under variable conditions. This variability is also obvious from OJIP curves sampled in different species (Ceppi 2010). It is not particularly representative for a photo-(electro)chemical reaction type. It has received ample application in quantitative descriptions of processes involved in bioreactor technology (Walas 1991). It is quite c different from the so-called Chapman-Richards sigmoid function *f*(*t*) = *A*[1 − exp(−kt)]^s^ in which k is a rate constant and s the variable sigmoidal factor that alters the steepness of the exponential rise at *s* = 1 (Joly and Carpentier 2009). This function has been applied in comparative MTF in WT and PSI mutants of Arabidopsis (Joly et al. 2010) with special emphasis on the I–P phase. The application in its present form however is hampered by the fact that the estimated values of the simulation parameters *s* and *k* cannot easily be related to measurable kinetic parameters or entities of the bioenergetic processes that are involved and operational during the IP phase of the fluorescence induction.

The disproportionally increased magnitude of the *F*_v_− decay component (*a*_4_ ~ 1.8, and rate *k*_4_ ~ (1 s)^−1^) (Fig. 9) as compared to $\Delta F_{\text{v}}^{\text{IP}}$ during the I–P phase in the light period between 50 and 500 ms (Figs. 8, 9), gives strong support for the earlier proposed hypothesis that the variable fluorescence (*F* ^CET^) in this phase is caused by a photo-electric stimulatory effect on the fluorescence yield. An effect of this kind comes into expression in the Boltzmann term $e^{{\left( {\psi_{0} - \psi } \right)}}$ which equals the ratio *k*_t_/*k*_−1_ of the energy transfer parameters for charge separation (*k*_t_) and –recombination (*k* _−1_) in the RC. An increase in the strength of an electric field and its associated potential *Ψ* at the charge-separated state of the RC at a constant value the redox potential *Ψ*_0_ of this state (with *Ψ*_0_, like *Ψ*, in units of the electrochemical entity RT/F ~ 25 mV at room temperature) will down-regulate the occupancy of the charge-separated state and consequently causes an increase in the fluorescence yield *Φ*_f_ of the antenna chlorophylls. This phenomenon shows the characteristics of what has been called non photochemical RC quenching (Ivanov et al. 2008). The expression for the fluorescence quantum yield *Φ*_f_ accounting for the three types of quenching has been derived (Bulychev and Vredenberg 2001; Vredenberg 2011)

9 $$\phi_{\text{f}} (\theta_{1} ,\theta_{2} ,\psi ) = \frac{1}{{1 + \frac{{k_{\text{w}} }}{{k_{\text{f}} }} + \frac{{[\theta_{1} (k_{\text{e}} + k_{\text{y}} ) + \theta_{2} k_{\text{y}} + k_{\text{d}} ]}}{{k_{\text{f}} N}}e^{{^{{(\psi_{o} - \psi )}} }} }}$$

in which probabilities of energy–dissipation in the antennas (*N* per RC) via fluorescence (*k*_f_,) and heat (*k*_w_), and probabilities of photochemical-(*k*_e_), photoelectrochemical trapping (*k*_y_) and non-radiative dissipation (*k*_d_) in RCs including (photo-)electric control and regulation via the Boltzmann term have come into expression. *θ*_1_ and *θ*_2_ are RC-fractions θ (0 ≤ *θ* ≤ 1) with unaffected (*θ*_1_) and acceptor side inhibited (*θ*_2_) charge stabilization, respectively. The difference in fluorescence yield of a closed (*θ*_1_,*θ*_2_) = (0,0) and open RC [(*θ*_1_,*θ*_2_) = (1,1)], according to Eq. 9, is dependent on the potential *Ψ*. It follows easily (see for a graphical illustration for instance Fig. 1 in (Vredenberg and Bulychev 2002) that for an open center [(*θ*_1_,*θ*_2_) = (1,1)], the increase in *φ*_f_(*Θ*_1_,*Θ*_2_,Δ*Ψ*) upon a distinct increase in *Ψ* (Δ*Ψ* > 0) is larger than for a closed RC [(*θ*_1_,*θ*_2_) = (0,0)]. A second conclusion is that the difference in fluorescence yield of an RC in the presence (Δ*Ψ* > 0) and absence of a potential change (Δ*Ψ* = 0) is higher in an open RC as compared to that in a closed one. Both conclusions have their counterparts in what is shown in Fig. 9 for the two major components of the *F*_v_ decay at 50 and 500 ms, i.e., at the I and P level, respectively. At the J-level where the RCs are nearly all closed *Θ*_1_ ~ *Θ*_2_ ~ 0 the (major) decay component, associated with the re-opening of RCs, is with rate constant *k*_3_ = *k*_−qbf_ = ~(50 ms)^−1^. The contribution of this component to the re-opening processes at the P-level is smaller, whereas that of the component with *k*_4_ = *k*_−IP_ ~ (1 s)^−1^ is considerably increased. Thus these results are in harmony with the hypothesis that the I–P part of the thermal JIP phase is caused by a (photo-) potential dependent stimulation of the fluorescence yield. The reversal of this potential in the dark, which might be considered as the release of the RC quenching is substantially slower than that of the photo-(electro) chemical quenching.

### A personal view

I started research in bioenergetics of photosynthesis in the young Biophysics Group of Lou Duysens at the University of Leiden, the Netherlands. In my PhD period during 1960–1965. I had the privilege to work in an inspiring scientific environment where novel ideas about the existence and properties of two interacting photochemical systems in algae, plants and isolated chloroplasts, and energy trapping in and closure of photosynthetic reaction centers were given a solid biophysical framework. Part of this work has been published in milestone papers (Duysens et al. 1961; Vredenberg and Duysens 1963; Duysens and Sweers 1963; van Grondelle and van Gorkom 2014). One of the starting points was focused on the relation between the RC closure and the increase in fluorescence yield. It was argued that photochemical conversion of either the primary donor P or primary acceptor, now known as Phe will lead to RC closure and subsequently to an increase in the fluorescence yield of the antenna chlorophyll. The role of the photochemical oxidation of the reaction center chlorophyll P (P890) in RC closure was demonstrated in bacteria from the associated increase in (bacterio-)chlorophyll fluorescence (Vredenberg and Duysens 1963; Vredenberg 1965). In algae and chloroplasts the fluorescence increase by PSII and its reversal by PSI was attributed to the photochemical conversion of a quencher *Q* and later argued to be identical to the primary quinone acceptor Q_A_ (Duysens and Sweers 1963). It is of interest to note, certainly in the 60 s, (i) a quencher was qualified by its unique property of causing RC closure upon its photochemical conversion and (ii) closure of RCs could exclusively be accomplished by photochemical conversion. Because of the relation between RC closure and increase in antenna chlorophyll fluorescence, the common opinion then has started to settle that a maximal fluorescence *F*_m_ is caused by 100 % photochemical reduction of Q_A_, or, cited from a recent review (Stirbet and Govindjee 2012) *‘full reduction of* Q_A_ *is required and sufficient for reaching F*_m_ *.’* This means, in terms of the original concept that full photochemical conversion of the quencher is required and sufficient for the closure of the reaction center. As a principal investigator at the Wageningen Center of Agro-Biological Research in the 70 s and late 60 s, I focused and performed experimental activities on active transport in plants and in particular on light-driven changes in transmembrane electrical potentials of green characean cells using micro-capillary glass electrodes. This gave me a view among others on the electrogenic properties of proton pumps in biological membranes (Vredenberg 1997). Owing to increased technical possibilities and application of patch-clamp techniques, these properties and light-induced effects were successfully studied in and across the thylakoid membrane of giant chloroplasts in *Peperomia metallica.* This research has been highly stimulated by frequent cooperation and joint research with Alexander Bulychev from Moscow State University, starting in 1975 and continued in the 80 s in the Photosynthesis group of the Department of Plant Physiology at the Wageningen University (WUR) with a number of PhD students (see http://www.rozenbergps.com/vredenberg/ under tab PhD Theses). For the understanding and able interpretation of electrical signals across the thylakoid membrane induced by one or more (repetitive) saturating single-turnover flashes (STF), I resumed in the mid-90 s the research on chlorophyll fluorescence (changes) under comparable experimental conditions. Since the mid-70 s I had followed the progress in this chlorophyll fluorescence area only at some distance. I was rather surprised to learn that the maximal fluorescence *F*_m_ was more or less dogmatically interpreted as the solid indicator of a 100 % reduction of Q_A_. This meant the exclusion of any effect in vivo of electrogenic events, for instance RC quenching associated with enhanced radical pair recombination, on RC closure and *F*_m_. The frequent research cooperation since my retirement in 2002 with Ondrej Prasil and coworkers from Trebon has greatly stimulated the final part of my research activities. These dealt with experiments on the fluorescence kinetics in algae, chloroplasts, and intact leaves during and after saturating µs-STFs and short pulses (sSPs) of variable duration and intensity. This paper is an example thereof. All these have given support for and strengthen the conviction that quantitative models for describing the variable chlorophyll fluorescence in relation to photosynthetic energy conversion should incorporate contributions of a second excitation of PSII and of RC quenching to the closure of the RC of this photosystem.

I consider this paper as the closure of a fascinating period in which I had the opportunity to give scientific contributions in the exciting field of photosynthesis research in particular in the area that is focused on the biophysical aspects of the primary and associated reactions in intact photosynthetic organisms ranging from bacteria to intact leaves. The monitoring of light-driven changes in the chlorophyll fluorescence yield has proven to be a sensitive and non-invasive experimental method to get a closer insight in the inner-sanctum of the complex machinery of the ongoing processes and reactions. Chlorophyll fluorescence indeed is, as the sub-title of a frequently cited book says, a signature of photosynthesis. However, for being a signature it demands that applications of the nowadays available fluorescence tools lead to the correct answers. Unfortunately, these demands are not always fulfilled and certainly not in the case of the interpretation of the maximal fluorescence yield F_m_ in a high intensity light pulse in relation to the properties of the closed state of the reaction center. As long as the debate whether or not the closing of a photosynthetic reaction center is exclusively dependent on the redox state of one or more fluorescence quenchers continues and has not led to a *communis* *opinio,* a large number of interpretations and conclusions on photosynthetic parameters are suspicious. I feel it of utmost and urgent importance that the debate is intensified and where needed is fed by new experiments that give added value for a rapid solution of the opposing and sometimes dogmatic views. I believe that the present results on the rate constants of processes that occur after reaction center closure in distinguishable phases of its re-opening in the dark will contribute to the enhancement and decisive phase of the debate. Not surprising my proclaim on the conclusion in the final debate is, in line with what has been expressed in earlier papers, that closing of an RC is ***not*** exclusively dependent on the photochemical reduction of Q_A_, or paraphrasing the statement in (Stirbet and Govindjee 2012), that ‘..*full reduction of* Q_A_ *is neither sufficient nor required for reaching Fm…’* Finally, I foresee that future research on the long-term kinetics of fluorescence induction in relation to that of photosynthetic processes will include a focus on (i) validation of the assumption that photochemical conversion of antenna fluorescence quenchers other than those bound to the RC leads to RC closure, (ii) the occurrence, strength, and effect of reaction center quenching in vivo, and (iii) the role of ATPases in acting as a proton leak for the proton motive force generated by the proton pumps generated in particular by cyclic electron transport (*F* ^CET^) around PSI. It is presumed that in particular the latter focus will lead to a better understanding of the kinetic profile of the Kautsky curve and its relation to the initial events of energy storage in the Calvin cycle.

## Abbreviations

ß: Fraction of Q_b_-nonreducing RCs
Δ*µ*_H_: Transmembrane proton motive force
DCMU: 3(3,4-Dichlorophenyl)-1,1-dimethylurea
dsq: Donor side quenching
*F*^PE^(*t*): Fluorescence emission at time *t*, relative to Fo, exclusively associated with release of photoelectrochemical quenching
*F*^PP^(*t*): Fluorescence emission at time *t*, relative to Fo, exclusively associated with release of photochemical quenching
*F*^CET^(*t*): Fluorescence emission at time *t*, relative to Fo, exclusively associated with photo-electric stimulation
*F*_0_: Fluorescence level of dark-adapted system with 100 % open RCs
*F*_m_: Fluorescence level of dark-adapted system with 100 % closed RCs after fluorescence saturating pulse excitation
$F_{\text{m}}^{\text{STF(SP)}}$: Fluorescence level after excitation with STF or SP, respectively of system in dark-adapted state
$F_{\text{m}}^{\text{PP}}$: Fluorescence level with 100 % semi-closed RCs after release of photochemical quenching
$F_{\text{ss}}^{\text{PP}}$: Steady state value in the light of the variable fluorescence associated exclusively with primary photochemical quenching
FIA: Fluorescence induction algorithm
*k*_AB_: Rate constant of ${\text{Q}}_{\text{A}}^{ - }$ oxidation
*k*_2AB_: Rate constant of oxidation of the double-reduced acceptor pair [PheQ_A_]^2−^ in reduced Q_B-_nonreducing RCs
*k*_L_: Excitation rate of photosystem in light pulse
*k*_qbf_: Rate constant of increase in variable fluorescence induced by *F* ^PE^ in 2–50 ms time domain
*k*_−qbf_: Rate constant of dark reversion of variable fluorescence in 2–50 ms time domain induced by *F* ^PE^
*k*_IP_: Rate constant of *F*_v_ fluorescence in 50–500 ms time domain induced by *F* ^CET^
*k*_-IP_: Rate constant determining the major decay component of *F*_v_ induced by *F* ^CET^
*nF*_v_: Maximal normalized variable fluorescence associated with 100 % photoreduction of Q_A_ of open RCs (*nF*_v_ = $F_{\text{m}}^{\text{PP}}$ = $F_{\text{m}}^{\text{STF}}$)
OEC: Oxygen evolving complex
Ph(e): Pheophytin, primary electron acceptor of PSII
PSII: Photosystem II
Q_A_: Primary quinone electron acceptor of PSII
Q_B_: Secondary quinone electron acceptor of PSII
*q*^dsq^: Fraction of PSII RCs in which photochemical quenching at acceptor and donor side is released
RC: Reaction center of photosystem
SP: Fluorescence saturating pulse with duration exceeding 250 ms
sSP: Short fluorescence excitation light pulse with duration between 0.25 and 500 ms
STF: Single-turnover flash (excitation)
TSTM: Three-state trapping model
*Y*_Z_: Secondary electron donor of PSII

## References

[CR1] Antal T, Rubin A (2008). In vivo analysis of chlorophyll a fluorescence induction. Photosynth Res.

[CR2] Belyaeva NE, Schmitt F-J, Steffen R, Paschenko VZ, Riznichenko GY, Chemeris YK, Renger G, Rubin AB (2008). PS II model-based simulations of single turnover flash-induced transients of fluorescence yield monitored within the time domain of 100 ns–10 s on dark-adapted Chlorella pyrenoidosa cells. Photosynth Res.

[CR3] Boisvert S, Joly D, Carpentier R (2006). Quantitative analysis of the experimental O-J-I-P chlorophyll fluorescence induction kinetics. Apparent activation energy and origin of each kinetic step. FEBS J.

[CR4] Bulychev AA, Vredenberg WJ (2001). Modulation of photosystem II chlorophyll fluorescence by electrogenic events generated by photosystem I. Bioelectrochem.

[CR5] Butler WL (1972). On the primary nature of fluorescence yield changes associated with photosynthesis. Proc Natl Acad Sci USA.

[CR6] Ceppi MG (2010) Paramètres photosynthétiques affectant le transport d’électrons é travers le pool de plastoquinone: la densite´ des photosystémes I, le contenu de chlorophylle et l’activité d’une plastoquinol-oxydase. PhD Thesis No 4175, University of Geneva, Geneva. Available at http://archive-ouverte.unige.ch/unige p. 5387

[CR7] Ceppi MG, Oukarroum A, Ciciek N, Strasser RJ, Schansker G (2011). The IP amplitude of the fluorescence rise OJIP is sensitive to changes in the photosystem I content of leaves: a study on plants exposed to magnesium and sulfate deficiencies, drought stress and salt stress. Physiol Plant.

[CR8] Chylla RA, Whitmarsh J (1989). Inactive photosystem ii complexes in leaves turnover rate and quantitation. Photosynth Res.

[CR9] Dinc E, Ceppi MG, Toth SZ, Bottka S, Schansker G (2012). The chl a fluorescence intensity is remarkably insensitive to changes in the chlorophyll content of the leaf as long as the chl a/b ratio remains unaffected. Biochim Biophys Acta.

[CR10] Duysens LNM, Sweers HE (1963) Mechanisms of the two photochemical reactions in algae as studied by means of fluorescence. In: Japanese society of plant physiologists. Studies on microalgae and photosynthetic bacteria, University of Tokyo Press, Tokyo, pp. 353–372

[CR11] Duysens LNM, Amesz J, Kamp BM (1961). Two photochemical systems in photosynthesis. Nature.

[CR12] Govindjee G, Papageorgiou GC, Govindjee (2004). Chlorophyll a fluorescence: a bit of basics and history. Chlorophyll a fluorescence: a signature of photosynthesis. Advances in photosynthesis and respiration.

[CR13] Guo Y, Tan J (2015). Recent advances in the application of chlorophyll a fluorescence from photosystem II. Photochem Photobiol.

[CR14] Ivanov AG, Sane PV, Hurry V, Öquist G, Huner NPA (2008). Photosystem II reaction centre quenching: mechanisms and physiological role. Photosynth Res.

[CR15] Joly D, Carpentier R (2009). Sigmoidal reduction kinetics of the photosystem II acceptor in intact photosynthetic materials during fluoerescence induction. Photochem Photobiol Sci.

[CR16] Joly D, Jemâa E, Carpentier R (2010). Redox state of the photosynthetic electron transport chain in wild-type and mutant leaves of Arabidopsis thaliana: impact on photosystem II fluorescence. J Photochem Photobiol, B.

[CR17] Kalaji HM, Goltsev V, Bosa K, Allakhverdiev SL, Strasser RT (2012). Experimental in vivo measurements of light emission in plants; a perspective dedicated to David Walker. Photosynth Res.

[CR18] Kautsky H, Hirsch A (1931). Neue Versuche zur Kohlensäureassimilation. Naturwiss.

[CR61] Klughammer C, Schreber U (2015) Apparent PS II absorption cross-section and estimation of mean PAR in optically this and dense suspensions of Chlorella. Photosynth Res 123:72–82 10.1007/s11120-014-0040-6 25218266

[CR19] Koblizek M, Kaftan D, Nedbal L (2001). On the relationship between the non-photochemical quenching of the chlorophyll fluorescence and the photosystem II light harvesting efficiency. A repetitive flash fluorescence study. Photosynth Res.

[CR20] Kramer DM, Johnson G, Kiirats O, Edwards GE (2004). New fluorescence parameters for the determination of QA redox state and excitation energy fluxes. Photosynth Res.

[CR21] Lavergne J, Leci E (1993). Properties of inactive photosystem II centers. Photosynth Res.

[CR22] Lazár D (2006). The polyphasic chlorophyll *a* fluorescence rise measured under high intensity of exciting light. Funct Plant Biol.

[CR23] Lazár D, Schansker G, Laisk A, Nedbal L, Govindjee (2009). Modeling of chlorophyll a fluorescence transients. Photosynthesis in silico: understanding complexity from molecules to ecosystems.

[CR24] Mauzerall D (1972). Light induced fluorescence changes in Chlorella, and the primary photoreactions for the production of oxygen. Proc Natl Acad Sci USA.

[CR25] Papageorgiou GC, Govindjee (2004). Chlorophyll a fluorescence: a signature of photosynthesis, advances in photosynthesis and respiration.

[CR26] Papageorgiou GC, Tsimilli-Michael M, Stamatakis K (2007). The fast and slow kinetics of chlorophyll a fluorescence induction in plants, algae and cyanobacteria: a viewpoint. Photosynth Res.

[CR27] Pospìŝil P, Dau H (2000). Chlorophyll fluorescence transients of Photosystem II membrane particles as a too for studying photosynthetic oxygen evolution. Photosynth Res.

[CR28] Robinson HH, Crofts AR (1983). Kinetics of the oxidation-reduction reactions of the Photosystem II quinone acceptor complex and the pathway of de-excitation. FEBS Lett.

[CR29] Samson G, Bruce D (1996). Origin of the low yield of chlorophyll fluorescence induced by single turnover flash in spinach thylakoids. Biochim Biophys Acta.

[CR30] Samson G, Prasil O, Yaakoubd B (1999). Photochemical and thermal phases of chlorophyll a fluorescence. Photosynthetica.

[CR31] Schansker G, Toth SZ, Strasser RJ (2005). Methylviologen and dibromothymoquinone treatments of pea leaves reveal the role of photosystem I in the Chl a fluorescence rise OJIP. Biochim Biophys Acta.

[CR32] Schansker G, Toth SZ, Strasser RJ (2006). Dark recovery of the Chl a fluorescence transient (OJIP) after light adaptation: the qT-component of non-photochemical quenching is related to an activated photosystem I acceptor side. Biochim Biophys Acta.

[CR33] Schansker G, Tóth SZ, Kovács L, Holzwarth AR, Garab G (2011). Evidence for a fluorescence yield change driven by a light-induced conformational change within photosystem II during the fast chlorophyll a fluorescence rise. Biochim Biophys Acta.

[CR34] Schansker G, Tóth SZ, Holzwarth AR, Garab G (2013). Chlorophyll a fluorescence: beyond the limits of the QA-model. Photosynth Res.

[CR35] Stirbet A (2013). Excitonic connectivity between photosystem II units: what is it, and how to measure it?. Photosynth Res.

[CR36] Stirbet A, Govindjee (2012). Chlorophyll a fluorescence induction: a personal perspective of the thermal phase, the J-I–P rise. Photosynth Res.

[CR37] Stirbet AD, Govindjee, Strasser BJ, Strasser RJ (1998). Chlorophyll *a* fluorescence induction in higher plants: modeling and numerical simulation. J Theor Biol.

[CR38] Strasser RJ, Srivastava A, Govindjee (1995). Polyphasic chlorophyll a fluorescence transient in plants and cyanobacteria. Photochem Photobiol.

[CR39] Strasser RJ, Tsimilli-Michael M, Srivastava A, Papageorgiou GC, Govindjee (2004). Analysis of the fluorescence transient. Chlorophyll a fluorescence: a signature of photosynthesis. Advances in Photosynthesis and Respiration.

[CR40] Suggett DJ, Prasil O, Borowitzka MA (2010). Chlorophyll a fluorescence in aquatic sciences. Methods and applications, developments in applied phycology.

[CR41] Tomek P, Ilik P, Lazár D, Stroch M, Naus J (2003). On the determination of QB-nonreducing photosystem II centers from chlorophylla fluorescence induction. Plant Sci.

[CR42] van Grondelle R, van Gorkom H (2014). The birth of the photosynthetic reaction center: the story of Lou Duysens. PRES. Photosynth Res.

[CR62] van Kooten O, Snel JFH (1990) The use of chlorophyll fluorescence nomenclature in plant stress physiology. Photosynth Res 25:147–150 10.1007/BF00033156 24420345

[CR43] Vredenberg WJ (1965) Spectrophotometric studies on primary and associated reactions in photosynthesis. PhD thesis University of Leiden (the Netherlands) p. 71

[CR44] Vredenberg WJ (1997). Electrogenesis in the photosynthetic membrane: fields, facts and features. Bioelectrochem.

[CR45] Vredenberg WJ (2000). A three-state model for energy trapping and chlorophyll fluorescence in photosystem II incorporating radical pair recombination. Biophys J.

[CR46] Vredenberg WJ, Papageorgiou GC, Govindjee (2004). System analysis of photoelectrochemical control of chlorophyll fluorescence in terms of trapping models of Photosystem II: a challenging view. Chlorophyll a fluorescence: a signature of photosynthesis, advances in photosynthesis and respiration.

[CR47] Vredenberg WJ (2008). Analysis of initial chlorophyll fluorescence induction kinetics in chloroplasts in terms of rate constants of donor side quenching release and electron trapping in photosystem II. Photosynth Res.

[CR48] Vredenberg WJ (2008). Algorithm for analysis of OJDIP fluorescence induction curves in terms of photo- and electrochemical events in photosystems of plant cells. Derivation and application. J Photochem Photobiol, B.

[CR49] Vredenberg WJ (2011). Kinetic analysis and mathematical modeling of primary photochemical and photoelectrochemical processes in plant photosystems. BioSys (Elsevier).

[CR50] Vredenberg WJ, Bulychev AA (2002). Photo-electrochemical control of photosystem II chlorophyll fluorescence in vivo. Bioelectrochemistry.

[CR51] Vredenberg WJ, Duysens LNM (1963). Transfer and trapping of excitation energy from bacteriochlorophyll to a reaction center during bacterial photosynthesis. Nature.

[CR52] Vredenberg WJ, Prasil O, Laisk A, Nedbal L, Govindjee (2009). Modeling of chlorophyll a fluorescence kinetics in plant cells: derivation of a descriptive algorithm. Photosynthesis in silico: understanding complexity from molecules to ecosystems.

[CR53] Vredenberg WJ, Prasil O (2013). On the polyphasic quenching kinetics of chlorophyll a fluorescence in algae after light pulses of variable length. Photosynth Res.

[CR54] Vredenberg WJ, Kasalicky V, Durchan M, Prasil O (2006). The chlorophyll a fluorescence induction pattern in chloroplasts upon repetitive single turnover excitations: accumulation and function of Q_B_-nonreducing centers. Biochim Biophys Acta.

[CR55] Vredenberg WJ, Durchan M, Prasil O (2007). On the chlorophyll fluorescence yield in chloroplasts upon excitation with twin turnover flashes (TTF) and high frequency flash trains. Photosynth Res.

[CR56] Vredenberg WJ, Durchan M, Prasil O (2009). Photochemical and photoelectrochemical quenching of chlorophyll fluorescence in photosystem II. Biochim Biophys Acta.

[CR57] Vredenberg WJ, Durchan M, Prasil O (2012). The analysis of PSII photochemical activity using single and multi-turnover excitations. J Photochem Photobiol, B.

[CR58] Vredenberg WJ, Kay J, Russoti R (2013) The instrumental implementation of a routine for quantitative analysis of photochemical-induced variable chlorophyll fluorescence in intact leaves: Properties and prospects. Poster (P78) at the 16th International Congress on Photosynthesis 11–16 August, St Louis, MO, USA

[CR59] Walas SM (1991) Modeling with differential equations in chemical engineering, vol 273. Butterworth-Heinemann, Boston, pp 4770–4777. ISBN ISBN 0706900127

[CR60] Zhu X-G, Govindjee Baker NR, deSturler E, Ort D, Long SP (2005). Chlorophyll a fluorescence induction kinetics in leaves predicted from a model describing each discrete step of excitation energy and electron transfer associated with photosystem II. Planta.

